# Environment predicts seagrass genotype, phenotype, and associated biodiversity in a temperate ecosystem

**DOI:** 10.3389/fpls.2022.887474

**Published:** 2022-08-04

**Authors:** Nahaa M Alotaibi, Emma J Kenyon, Chiara M Bertelli, Rahmah N Al-Qthanin, Jessica Mead, Mark Parry, James C Bull

**Affiliations:** ^1^Department of Biosciences, Swansea University, Swansea, United Kingdom; ^2^Department of Biology, Princess Nourah bint Abdulrahman University, Riyadh, Saudi Arabia; ^3^Department of Biology, King Khalid University, Abha, Saudi Arabia; ^4^Ocean Conservation Trust, National Marine Aquarium, Plymouth, United Kingdom

**Keywords:** microsatellites, population genetics, *Zostera marina*, seagrass, coastal resilience

## Abstract

Coastal vegetative ecosystems are among the most threatened in the world, facing multiple anthropogenic stressors. A good example of this is seagrass, which supports carbon capture, coastal stabilization, and biodiversity, but is declining globally at an alarming rate. To understand the causes and consequences of changes to these ecosystems, we need to determine the linkages between different biotic and abiotic components. We used data on the seagrass, *Zostera marina*, collected by citizen scientists across 300 km of the south coast of the United Kingdom as a case study. We assembled data on seagrass genotype, phenotype, infauna, and associated bathymetry, light, sea surface temperature, and wave and current energy to test hypotheses on the distribution and diversity of this temperate sub-tidal ecosystem. We found spatial structure in population genetics, evident through local assortment of genotypes and isolation by distance across a broader geographic scale. By integrating our molecular data with information on seagrass phenotype and infauna, we demonstrate that these ecosystem components are primarily linked indirectly through the effects of shared environmental factors. It is unusual to examine genotypic, phenotypic, and environmental data in a single study, but this approach can inform both conservation and restoration of seagrass, as well as giving new insights into a widespread and important ecosystem.

## Introduction

Increasing urbanization and resource exploitation, along with global climate change, is reducing resilience and accelerating loss of important coastal habitats and ecosystems, such as mangroves, salt marsh, and seagrass (Waycott et al., 2009; Silliman, 2014; Spivak et al., 2019; Stafford et al., 2021). Typical of these, seagrasses underpin numerous ecosystem services, including blue carbon, coastal stabilization, and supporting biodiversity of intrinsic value as well as critical to numerous commercial fisheries (Fourqurean et al., 2012; Kerr, 2017; Röhr et al., 2018; Unsworth et al., 2019). Seagrass declines in recent decades have been substantial, with greater than 29% loss estimated globally since the 1980s (Waycott et al., 2009), and even great declines found in many regions; for example, over 60% loss estimated in Swedish waters (Baden et al., 2003) and 39% loss reported in United Kingdom waters (Green et al., 2021), over the same time period.

The causes for these losses come from many sources: local environmental changes such as eutrophication, pollution, habitat loss or disturbance, and changes in resource-consumer interactions (Short et al., 2006), as well as potentially from breakdown of regional connectivity (Kendrick et al., 2017). In addition, numerous feedbacks are known to be at play in seagrass ecosystems, e.g., through self-facilitation as a result of stabilizing sediment and increasing water clarity (Adams et al., 2016), inbreeding depression as declining populations suffer accelerated losses due to the resulting lack of genetic diversity (Maxwell et al., 2017), or metapopulation Allee effects (Amarasekare, 1998) as loss of local populations weakens connectivity at the broader scale, diminishing metapopulation resilience observed in numerous seagrasses (Rozenfeld et al., 2008; Lobelle et al., 2013; Grech et al., 2018; Jackson et al., 2021).

Since this range of pressures acts through different processes and mechanisms on different ecosystem properties, it is increasingly well understood that assessment of seagrass population status cannot rely on any single metric (Unsworth et al., 2015), and several studies have conducted multivariate assessments of resilience in seagrass (Jones and Unsworth, 2016; Jahnke et al., 2020; Bertelli et al., 2021; Krumhansl et al., 2021) and other coastal vegetative ecosystems (Battisti et al., 2020). However, there is now a need to advance this multivariate bioindicator approach, to understand the connections between components of seagrass ecosystems, linking environmental variables, seagrass genotype, seagrass phenotype, and associated biodiversity.

Our overall aim here was to understand the connections between key biotic and abiotic components in a seagrass ecosystem. While traditional species-habitat association models focus on correlations between presence of a target species and environmental covariates (Matthiopoulos et al., 2020), we wanted to understand the associations between environmental factors and multiple system components simultaneously. To achieve this, our specific objectives were to (1) quantify spatial population genetic structure in *Zostera marina* meadows across the southwest of the United Kingdom, based on hierarchical analysis of molecular variance (AMOVA), isolation by distance (IBD), and clustering methods, (2) use a mixed effects modeling approach to test hypotheses on environmental predictors of *Z. marina* population genetic variation as a first step toward understanding habitat suitability associated with genetic resilience in seagrass, and (3) develop a structural equation model (SEM) linking environmental drivers, seagrass genotype, seagrass phenotype, and major seagrass-associated faunal groups.

This study is correlative, rather than mechanistic, and focusses on genetic diversity measured through neutral genetic markers, accessible and affordable to a citizen science initiative. However, observations and analysis from this established seagrass ecosystem across a widespread natural environment complements and potentially can be used to validate more manipulative, experimental studies. Insight from this type of integrated analysis of system components is important to the development of habitat management, restoration, and policy; particularly as nature-based solutions are sought in coastal settings (Kumar et al., 2021; Seddon et al., 2021).

## Materials and methods

This study uses (1) novel seagrass population genetic data (Supplementary material), (2) seagrass phenotype and associated fauna data freely available as supplementary material associated with Smale et al. (2019), and (3) environmental data obtained from the European Marine Observation and Data Network (EMODnet) seabed habitats portal^1^ and Copernicus Marine Environment Monitoring Service.^2^

### Study area

**Figure 1:**
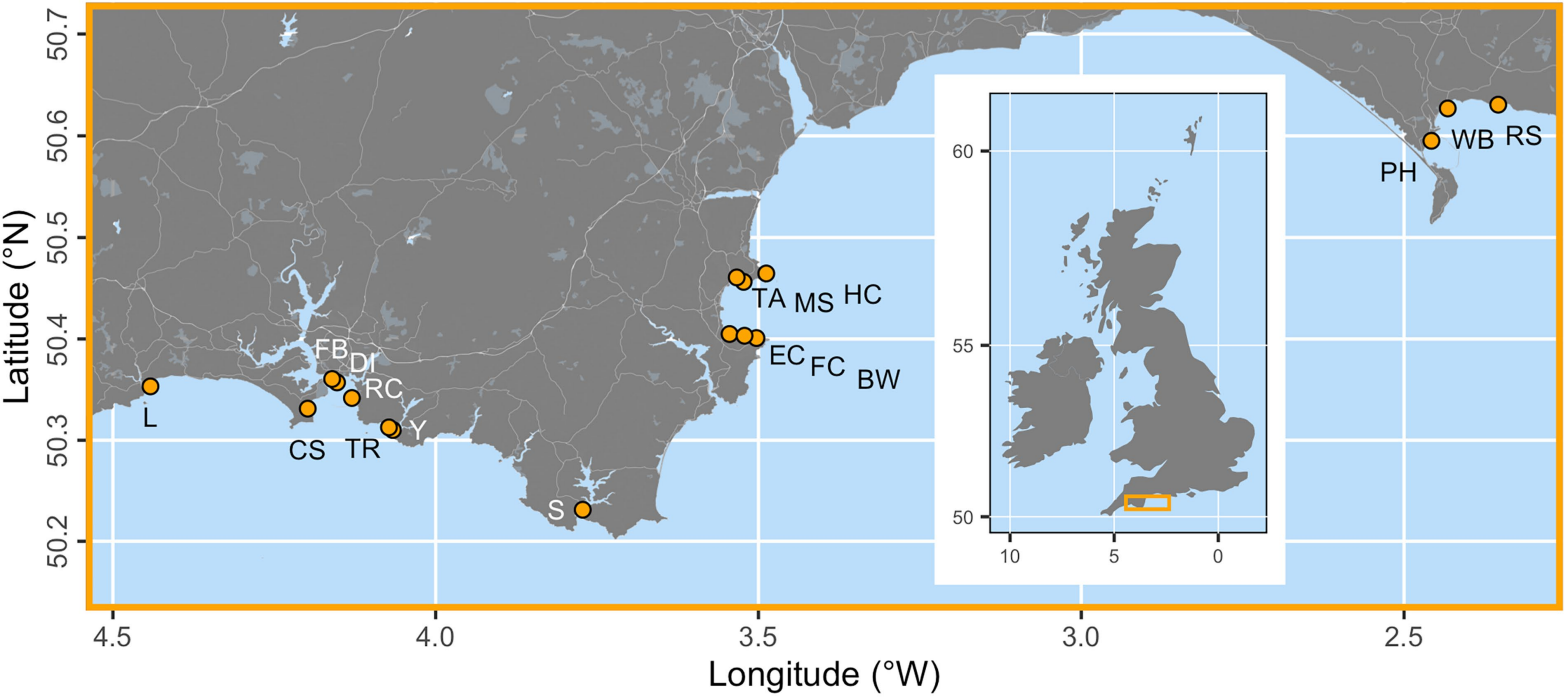
The locations of 17 seagrass study sites (orange points) across the south coast of England. Sampling locations are Looe (L), Cawsands (CS), Firestone Bay (FB), Drake’s Island (DI), Ramscliffe (RC), Tomb Rock (TR), Cellars Cove at Yealm (Y), Salcombe (S), Elberry Cove (EC), Fishcombe Cove (FC), Brixham Breakwater (BW), Torre Abbey (TA), Millstones Bay (MS), Hope Cove (HC), Portland Harbour (PH), Weymouth Bay (WB), and Ringstead Bay (RS). The inset map of the British Isles shows the placement of the main map as an orange box.

Seventeen locations were assessed along a c. 300 km stretch of the south coast of England (Figure 1). These sites were selected at random from a larger pool (Smale et al., 2019). Survey locations were situated c. 1–5 m depth (see Figure 1).

### Sampling methods

Surveys were conducted by trained volunteer divers during August of 2016 and 2017 as part of the Community Seagrass Initiative (CSI), a citizen science project led by the National Marine Aquarium and contributing partners, funded by the United Kingdom Heritage Lottery Fund. Full survey details are published in Smale et al. (2019). Seagrass presence/absence, as well as shoot density, was estimated by placing 15 quadrats (0.25 m^2^) along each of a series of predefined transects, parallel to the shore, varying between 50 and 150 m, dependent upon the size of the seagrass meadow. Abundance of nine faunal groups were also assessed within these quadrats: ascidians, bryozoans, cnidarians, crustaceans, echinoderms, fish, molluscs, sponges, and worms. The emphasis was on broad taxonomic classification that could be reliably identified by trained volunteers (Smale et al., 2019). While this simplification did not allow for a robust quantification of biodiversity, it did allow for many samples to be collected across large spatial scales with a high degree of confidence. Fauna associated with both *Zostera marina* and the underlying substratum were recorded. All quadrats were photographed *in situ* and any uncertainties with faunal identification were subsequently addressed through consultation with relevant experts at the National Marine Aquarium, Plymouth.

For the purposes of the current study the transect quadrats were subsampled haphazardly such that one *Z. marina* shoot was collected from each of c. 20 quadrats, at least 2 m apart, from each survey location, cleaned, and briefly air dried, then individually bagged before being transferred to Swansea University for molecular analysis. A total of 307 samples were collected across the 17 locations. Samples from 2016 and 2017 were pooled at each location.

### Molecular methods

Frozen samples were ground using a Precellys Ceramic 1.4/2.8 mm kit. DNA was extracted using Qiagen DNEasy Plant kits, with purity and concentration assessed by spectrophotometry. We used a published panel of 15 microsatellites (Supplementary Table 1), labeled and pooled as in their original publication (Oetjen et al., 2010). PCR amplification was accomplished according to the manufacturer’s protocols using Qiagen Type-it Microsatellite PCR kits. PCR products were sent to the Institute of Biological, Environmental, and Rural Sciences (IBERS), Aberystwyth University, United Kingdom, to obtain read lengths. Fragment Analysis was performed on an ABI 3730 DNA Analyser, using a 48 capillary Array (50 cm length) and POP-7 Polymer. Samples were run using Run Module: GeneMapper50_POP7_1, Dye set-G5.

### Population genetic analysis

Microsatellite DNA fragment lengths were used as the basis of allele scoring. This was achieved using the R package Fragman v1.0.9, automatically with default settings and confirmed through manual inspection (Covarrubias-Pazaran et al., 2016). We visualized the scoring and binning of microsatellite alleles using the R package MsatAllele v1.0 (Alberto, 2009). This permitted the identification of monomorphic and polymorphic loci, as well as providing a further check of allele identifiability at the population level. Presence of null alleles was assessed using FreeNA software (Chapuis and Estoup, 2007).

We assessed the probability of two or more samples coming from independent reproductive events using *P_SEX_* (Arnaud-Haond et al., 2007), removing samples where *p* < 0.05. Using this slightly reduced dataset, we quantified the observed number of multilocus genotypes (MLG) at each survey site using the R package Poppr v2.8.1 (Kamvar et al., 2014). We also calculated a common measure of genotypic diversity: clonal richness, R = (MLG – 1)/(*N* – 1), where *N* is the sample size (Dorken and Eckert, 2001). To further evaluate microsatellite diversity, we calculated the average number of alleles per polymorphic locus (Ar, allelic richness) and observed heterozygosity compared to expected levels assuming Hardy–Weinberg equilibrium (Fis) at each of our 17 locations, using Poppr v2.8.1.

To assess the population genetic structure of *Z. marina* across the south coast of the UK, initially a hierarchical analysis of molecular variance (AMOVA) was performed using Poppr v2.8.1, with statistical significance assessed through randomization testing with 999 permutations. Pairwise estimates of Fst were also calculated and compared against pairwise geographic distance between locations to test the hypothesis of isolation by distance (Rousset, 1997). We calculated the shortest at-sea distance (i.e., excluding land) between the 17 seagrass sites, using the R package gDistance v1.2–2 (van Etten, 2017). We tested the statistical significance of the correlation between genetic and geographic pairwise distances using a Mantel test with 999 permutations (Mantel, 1967). Finally, population structure was assessed by cluster analysis. K clusters were assessed using the cross-entropy criterion (Frichot and François, 2015) to identify the most likely number of genetic clusters, for K = {1, …, 5}. Proceeding with the optimal K value (minimizing cross-entropy), we used a sparse non-negative matrix factorization algorithm (snmf function in the freely available LEA R package; Frichot and François, 2015) that produces output in the style of the widely used STRUCTURE approach, using the default 200 iterations. This assigns a probability to every individual sampled of belonging to each of the proposed K clusters (the admixture coefficient), typically viewed as a stacked bar chart.

### Environmental data acquisition

Hydrographical data were gathered from EMODnet^3^ and included kinetic energy at the seabed due to waves (KeW) and seabed kinetic energy due to currents (KeC). Since *in situ* light data were not available, photosynthetically active radiation (PAR) at the seabed, sourced from EMODnet, was used as a proxy. Additionally, sea surface temperatures (SST) were obtained from Copernicus Marine Environment Monitoring Service.^4^ We selected monthly mean SST for March when seed germination and seedling emergence takes place and August for peak biomass and seed production (Sand-Jensen, 1975; Orth and Moore, 1986; Blok et al., 2018). Since genetic samples were collected across 2016 and 2017, we assembled SST data for both years. Variance inflation factor analysis (Naimi et al., 2014) indicated that the 2 years were highly correlated and that 2016 should be retained over 2017. For each location, the three grid squares (0.3 km resolution) closest to the survey position that contained environmental data were averaged to give an overall value. All data were clipped to the same geographical coordinates for 17 sites using the Spatial Analyst 9.3 extension from ArcGIS 9.3 software (ESRI). In addition, seabed depth was obtained from dive computer records, corrected for tide at the time of the dive using the Imray Tides Planner App. Depths were recorded by all pairs of divers and the median depth for each location used here.

### Statistical modeling

We tested hypotheses on the relationships between environmental and seagrass genotypic data using generalized linear mixed modeling. We used the Template Model Builder (TMB) approach (Magnusson et al., 2017) to construct statistical models able to incorporate (1) environmental covariates as fixed effects, (2) spatial coordinates as either fixed effects or a spatially autocorrelated covariance matrix between spatial random effects, and (3) non-Gaussian error distributions.

Separate statistical models were developed for different response variables: allelic richness (Ar), clonal richness (R), heterozygosity (Fis), and the probability of samples belonging to each of the proposed genetic clusters. Allelic richness was modelled using a gamma error distribution and log link function. Clonal richness is a proportion, bounded by 0 and 1, so was modelled using a beta error distribution and logit link function. The distribution of Fis values [1 – (Hobs/Hexp)] has an upper limit of 1 (no heterozygotes observed) but can be negative and is (theoretically) unbounded at its lower limit. Therefore, we transformed Fis into 1 – Fis (so mapping the 1 boundary to 0, and negative values become positive) and modelled this using a gamma distribution and log link function. Probabilities of belonging to proposed clusters must sum to 1 and were modelled using Dirichlet distributions and logit link function with the DirichletReg R package (Maier, 2014).

Nonlinear environmental fixed effects (Depth, KeW, KeC, PAR, March and August SST) were modelled with cubic base splines within the linear statistical model using the bs function of the splines R package (R Core Team, 2020). By way of validation, environmental variables were also fitted as additive linear and quadratic terms, but we preferred the splines approach for added flexibility and results were not qualitatively different to using quadratic regression. With the exception of the Dirichlet regression, models were run using the glmmTMB R package (Bolker, 2016; Magnusson et al., 2017). Models with different combinations of fixed environmental effects and/or spatial autocorrelation were compared using AIC modified for small sample sizes: the AICc function within the AICcmodavg R package (Mazerolle and Mazerolle, 2017).

### Structural equation modeling

We undertook a structured regression analysis to test hypotheses on linkages between the variables assembled in this study. This was conducted through structural equation modeling (SEM), using the sem R package (Fox, 2006). The pipeline we developed comprised:

1. Allocating all variables to one of four conceptual groups: “environment,” “seagrass genotype,” “seagrass phenotype,” or “associated fauna.”
2. We then performed pairwise Spearman rank correlation between all variables, filtering out those which did not show a statistically significant correlation (at the 5% level) and correlation coefficient, *r* > 0.5, with a variable from one of the other conceptual groups in step 1. Where variables were highly correlated (*r* > 0.5) with another variable in the same conceptual group, we performed a variance inflation factor (VIF) analysis and removed the variable with the higher VIF.
3. Next, we hypothesized whether a direction of causality could be assumed (⇨) or not (⇔) and proposed the model structure: “environment” ⇨ “seagrass genotype,” “environment” ⇨ “seagrass phenotype,” “environment” ⇨ “associated fauna,” “seagrass genotype” ⇨ “seagrass phenotype,” “seagrass genotype” ⇔ “associated fauna,” and “seagrass phenotype” ⇔ “associated fauna”.
4. Within the framework proposed in step 3, we populated the SEM with the variables retained after step 2.

The resulting SEM was assessed for goodness of fit using the model χ^2^ value, the Goodness of Fit Index (GFI, analogous to R^2^), Root Mean Square Error of Approximation (RMSEA), and Comparative Fit Index (CFI). Statistically significant associations (at the 1% level) were retained.

Where statistical analysis was performed using R, this was run in version 4.0.2 (R Core Team, 2020).

## Results

### Population genetic structure

The novel data generated for this study were population genetic. A total of 307 *Zostera marina* leaf samples were genotyped at 15 microsatellite loci (Supplementary Table 1). Four loci (CL11Contig1, ZME06302, ZMC19062, and ZME02369) were found to be monomorphic. These four loci were removed from all subsequent analyses, leaving a total of 11 informative markers: CL766Contig1 (4 alleles), CL559Contig1 (5 alleles), ZME02125 (3 alleles), ZMF02381 (7 alleles), CL202Contig1 (4 alleles), CL380Contig1 (9 alleles), CL805Contig1 (3 alleles), CL172Contig1 (5 alleles), CL53Contig1 (4 alleles), ZMC05062 (5 alleles), and ZME05315 (5 alleles). We quantified departure from Hardy–Weinberg equilibrium for all loci, across all sites in Supplementary Figure 1. Only three loci were found to substantially deviate from Hardy–Weinberg equilibrium at the individual location level: CL766Contig1, CL53Contig1, and ZMC05062.

While our sampling regime made re-sampling the same genet unlikely within a location and near impossible between locations, we accounted for this potential using the *P_SEX_* method, at a threshold of *p* = 0.05. Structuring this analysis by location, we removed 18 individuals (from 307), leaving a sample size of 289 individuals. The distribution of removed individuals across locations is shown in Supplementary Figure 2. The majority of removed samples were at Fishcombe Cove (FC), Brixham Breakwater (BW) in the Torbay area.

Overall, we found high levels of clonal richness, R, with the lowest value being 0.67 at Brixham Breakwater (BW) and R = 1 at five of the 17 locations (Table 1), indicating all samples were different multilocus genotypes at those locations. Allelic richness, Ar, ranged from 1.64 at Ringstead Bay (RS) to 2.55 at Salcombe (S) and Millstones Bay (MS). Finally, Fis was positive at all locations, indicating less observed heterozygosity than expected, ranging from 0.06 in Portland Harbour (PH) to 0.558 at Ramscliffe (RC).

We initially assessed population genetic structure using Analysis of Molecular Variance (AMOVA, Table 2). There was statistically significant variation at all levels, with comparable levels of variation between the 17 locations (SD = 0.78) and within samples (SD = 0.85), but lower variation between samples within sites (SD = 0.29). This equates to 20.2% of observed variation being attributed to differences between the 17 locations, with the remaining 79.8% accounted for within locations.

The hypothesis of isolation by (sea) distance was supported (r = 0.368, *p* = 0.001, Mantel test with 999 permutations). However, we found that square root distance was a better fit than untransformed distance (ΔAICc = 2.83, linear models), suggesting that the change in pairwise genetic distance with increasing geographic distance is strongest over the scale of kilometers to tens of kilometers, plateauing over longer ranges (Supplementary Figure 3).

**Figure 2:**
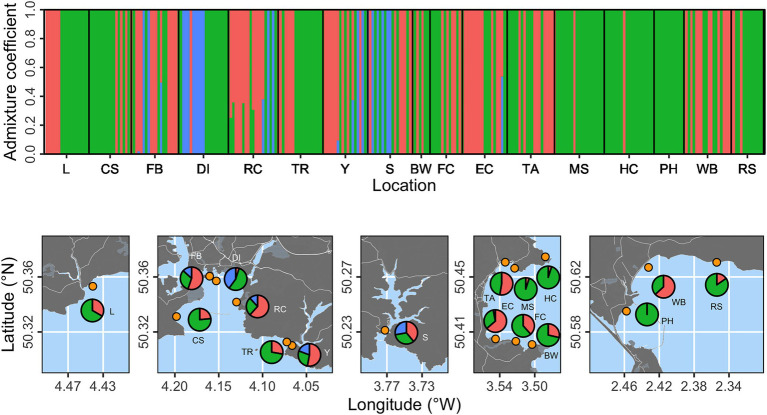
Population structure analysis based on three proposed genetic clusters. The top panel shows admixture coefficients indicating the probability of individual samples belonging to each of the three clusters (here shown as red, green, or blue). Bottom panels show location level summaries of admixture coefficients at each of the 17 sampling locations (orange points).

Cluster analysis was performed using a proposed three genetic clusters (based on cross-entropy scores for 1–5 populations) and presented at the individual and location levels (Figure 2). Individuals were found with high probability (*p* > 0.99) of belonging to each of the three clusters. Two clusters (shown as red and green in Figure 2) were substantially more prevalent than the third (blue in Figure 2). The “blue” cluster was only evident at five locations (FB, DI, RC, Y, and S), with the potential exception of a single individual with 0.6 probability of belonging to the “blue” cluster at Elberry Cove (EC). We summarized proportional prevalence at the location level in Figure 2, which highlighted that the “blue” cluster was strongly associated with more estuarine locations, rather than open coastline.

**Table 1:** *Zostera marina* population genetic parameters and associated environmental parameters from 17 sampling locations across the south of England.

Site	Lon.	Lat.	*N*	*N*_adj_	MLG	R	Ar	Fis	D	KeW	KeC	PAR	Mar	Aug
L	4.442	50.353	18	18	14	0.76	2.18	0.338	3.57	477	6.38	23.3	9.50	15.0
CS	4.198	50.331	17	17	17	1.00	2.18	0.255	1.64	205	8.98	14.0	9.37	15.7
FB	4,160	50.361	20	19	17	0.89	2.18	0.291	2.75	24.4	3.31	0.13	9.37	15.7
DI	4.153	50.357	20	20	20	1.00	2.27	0.378	1.42	120	3.16	5.06	9.37	15.7
RC	4.130	50.342	20	20	17	0.84	2.00	0.558	3.97	47.5	2.90	3.42	9.37	15.7
TR	4.072	50.313	19	18	17	0.94	1.91	0.400	1.37	1,970	17.1	5.58	9.37	15.7
Y	4.066	50.310	20	18	17	0.94	2.36	0.528	3.07	1,030	18.4	11.9	9.37	15.7
S	3.772	50.231	20	18	18	1.00	2.55	0.383	2.91	250	29.2	4.88	9.32	15.5
BW	3.503	50.401	12	7	5	0.67	1.82	0.470	1.06	0.44	29.5	12.1	9.16	14.8
FC	3.522	50.403	18	13	11	0.83	1.91	0.309	1.51	0.73	11.9	8.20	9.16	14.8
EC	3.545	50.505	18	18	18	1.00	2.18	0.341	4.00	0.74	10.8	10.2	9.16	14.8
TA	3.533	50.461	20	19	18	0.94	2.36	0.444	3.26	18.5	4.69	4.41	9.16	14.8
MS	3.523	50.456	20	20	18	0.89	2.55	0.058	3.61	20.3	5.71	3.75	9.16	14.8
HC	3.488	50.456	20	20	20	1.00	2.00	0.170	1.87	32.3	9.66	7.73	9.06	14.9
PH	2.458	50.595	13	12	10	0.82	1.73	0.060	1.87	3.34	27.9	9.38	8.67	14.8
WB	2.432	50.627	19	19	17	0.88	2.18	0.395	3.01	29.7	12.6	2.76	8.61	15.0
RS	2.354	50.631	13	13	10	0.75	1.64	0.260	1.03	375	24.0	3.75	8.61	15.0

Longitude and Latitude shown as decimal degrees (West and North respectively). *N*, number of samples; *N*_adj_, number of samples following *P_SEX_* adjustment; MLG, multilocus groups; R, clonal richness; Ar, allelic richness; Fis, heterozygosity relative to expectation; D, depth (m); KeW, kinetic energy due to waves (N m^2^ s^−1^); KeC, kinetic energy due to currents (N m^2^ s^−1^); PAR, photosynthetically available radiation at the seabed (mol. photons m^−2^ day^−1^), Mar, March 2016 sea surface temperature (°C); Aug, August 2016 sea surface temperature (°C).

**Table 2:** Analysis of molecular variance (AMOVA).

	Df	SS	MS	SD	% SD	*p*-value
Between locations	16	491	30.7	0.78	20.2	<0.001
Between samples	272	1,130	4.15	1.07	27.5	<0.001
Within samples	289	585	2.02	2.02	52.3	<0.001
Total	577	2,206	3.82	3.87	100	<0.001

SD shows the cumulative amount of total variation attributed to the levels of population structure. Statistical significance was assessed using randomization testing with 999 permutations. Between locations refers to the 17 sampling locations. Between samples refers to the c.20 quadrats sampled at each location (one leaf per quadrat). Within samples refers to differences between alleles of each leaf sample in this diploid species.

### Environmental predictors of population genetics

**Figure 3:**
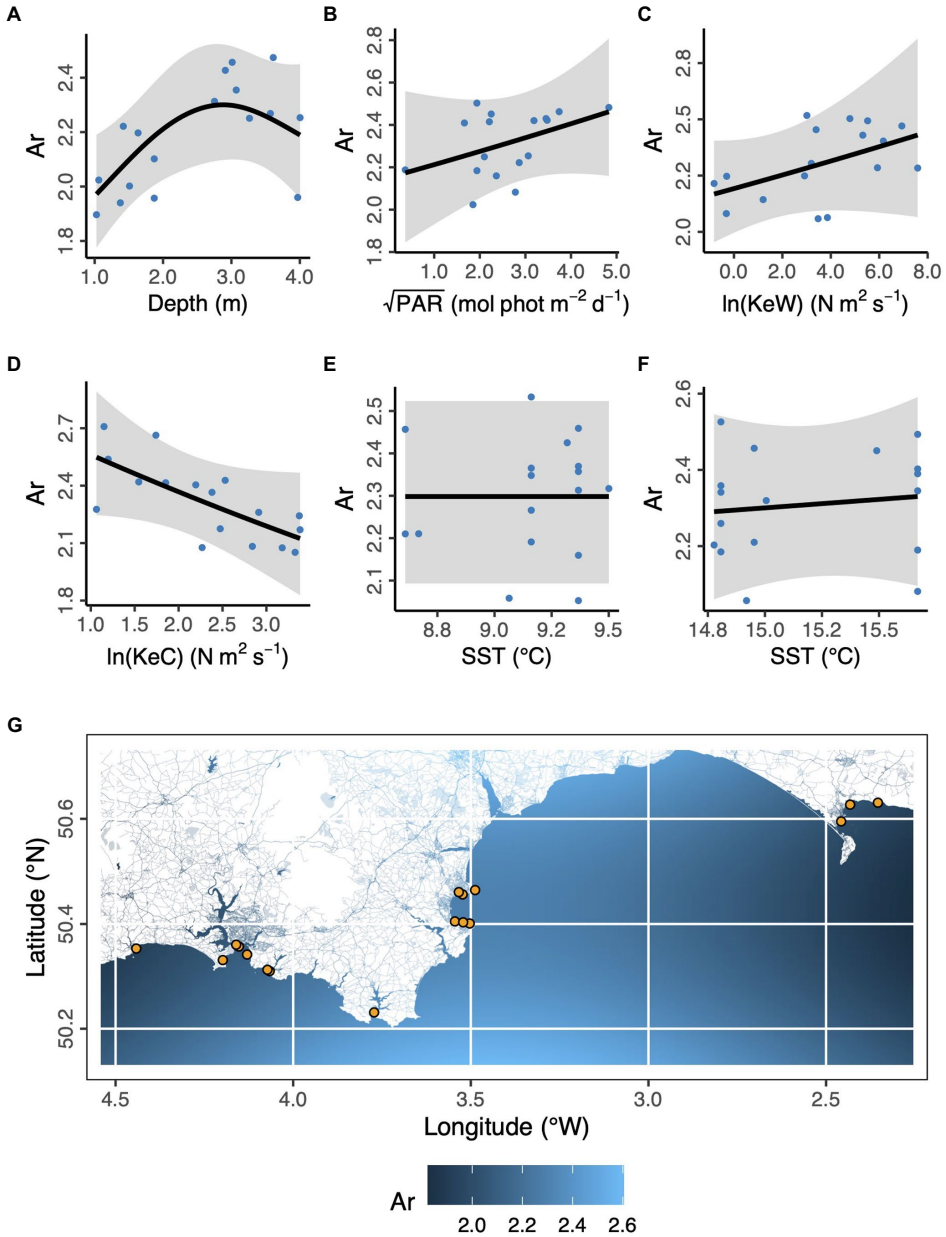
Environmental and geographical predictors of *Zostera marina* allelic richness, Ar, at 17 locations (orange points in panel **G**) across the south coast of England. **(A)** Depth is below chart datum. **(B)** PAR is photosynthetically active radiation at the seabed. KeW **(C)** and KeC **(D)** are kinetic energy, associated with waves and currents, respectively. SST is sea surface temperature in March **(E)** and August **(F)** 2016. Shaded ribbons show 95% confidence intervals and blue points are partial residuals. **(G)** The background color scheme represents fitted estimates of Ar in geographic space. Empirical values are shown in Table 1.

In the case of allelic richness, Ar, (Figure 3) geographic space (longitude, latitude) was not statistically significant as fixed effects (Longitude, *F* = 0.902, *p* = 0.107; Latitude, *F* = 0.270, *p* = 0.185; Longitude × Latitude, *F* < 0.001, *p* = 0.534). We also found modeling space as autocorrelated random effects resulted in a worse model than no spatial structure (ΔAICc = 31.1). However, in ecological niche space (Depth, PAR, KeW, KeC, SST), we found an increase in Ar with increasing depth, plateauing by around 3 m below chart datum (*F* = 3.53, *p* = 0.030). We found no other statistically significant relationships between Ar and environmental covariates: light (PAR, *F* = 1.08, *p* = 0.326), kinetic energy due to waves (KeW, *F* = 1.25, *p* = 0.292), kinetic energy due to currents (KeC, *F* = 2.87, *p* = 0.124), and sea surface temperature (March SST, *F* < 0.001, *p* = 0.644; August SST, *F* = 0.073, *p* = 0.270).

When we modelled clonal richness, R, we found that longitude and latitude were not statistically significant as fixed effects (Longitude, χ^2^ < 0.001, *p* = 0.638; Latitude, χ^2^ < 0.001, *p* = 0.905; Longitude × Latitude, χ^2^ < 0.001, *p* = 0.669), and that the algorithm would not converge with spatially autocorrelated random effects, likely due to lack of overall variation in our clonal richness values. Therefore, we concluded there were no substantial spatial influences on R. We also found none of our environmental predictors explained a statistically significant amount of deviance (Depth, *F* = 0.003, *p* = 0.960; PAR, *F* = 0.045, *p* = 0.832; KeW, *F* = 0.032, *p* = 0.858; KeC, *F* = 0.010, *p* = 0.920; March SST, *F* < 0.001, *p* = 0.604; August SST, *F* < 0.001, *p* = 0.719). Empirical values are shown in Table 1 and graphical relationships between environmental and geographical predictors of *Z. marina* clonal richness are shown in Supplementary Figure 4.

Similarly, with Fis we found that longitude and latitude were not statistically significant as fixed effects (Longitude, *F* < 0.001, *p* = 0.991; Latitude, *F* < 0.001, *p* = 0.957; Longitude × Latitude, *F* < 0.001, *p* = 0.763). We also found modeling space as autocorrelated random effects resulted in a worse model than no spatial structure (ΔAICc = 22.7). Again, we concluded there were no substantial spatial effects on Fis. Also as with R, we found none of our environmental predictors explained a statistically significant amount of deviance in Fis (Depth, *F* = 0.901, *p* = 0.362; PAR, *F* = 0.113, *p* = 0.743; KeW, *F* = 0.072, *p* = 0.794; KeC, *F* = 0.123, *p* = 0.733; March SST, *F* < 0.001, *p* = 0.739; August SST, *F* = 1.12, *p* = 0.100). Empirical values are shown in Table 1 and graphical relationships between environmental and geographical predictors of *Z. marina* Fis are shown in Supplementary Figure 5.

**Figure 4:**
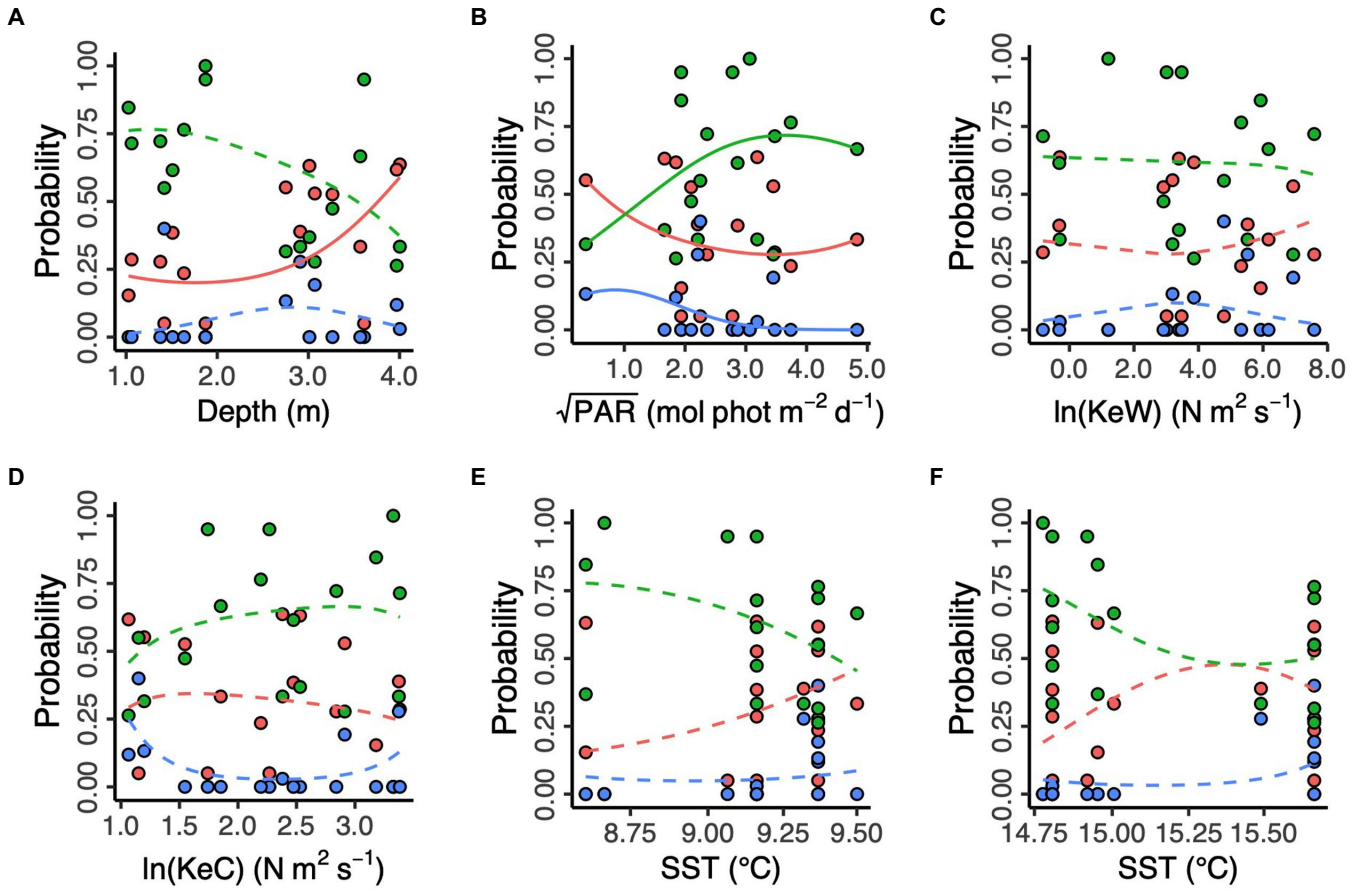
Environmental predictors of three putative *Zostera marina* clusters (“red,” “green,” and “blue”) in 17 locations across the south coast of England. **(A)** Depth is below chart datum. **(B)** PAR is photosynthetically active radiation at the seabed. KeW **(C)** and KeC **(D)** are kinetic energy, associated with waves and currents, respectively. SST is sea surface temperature in March **(E)** and August **(F)** 2016. Solid lines show statistically significant relationships (*p* < 0.05), with non-significant relationships shown as dashed lines.

Additionally, we modelled the effects of spatial and environmental predictors on the relative frequencies of each of the three proposed genetic clusters (Figure 4). We found no effect of longitude and latitude (χ^2^_df = 9_ = 12.39, *p* = 0.192, likelihood ratio test). Prevalence of the “red” cluster significantly increased with increasing depth (“Red,” χ^2^_df = 2_ = 7.13, *p* = 0.028). However, depth did not have a statistically significant effect on prevalence of the “green” and (estuary-associated) “blue” clusters (“Green,” χ^2^_df = 2_ = 4.46, *p* = 0.108; “Blue,” χ^2^_df = 2_ = 1.35, *p* = 0.509). Light (PAR) had a statistically significant effect on all three clusters (“Red,” χ^2^_df = 2_ = 42.1, *p* < 0.001; “Green,” χ^2^_df = 2_ = 38.2, *p* < 0.001; “Blue,” χ^2^_df = 2_ = 37.2, *p* < 0.001), with the “green” cluster more likely to be found in higher light conditions and the “red” and “blue” clusters more likely in lower light conditions. Kinetic energy did not have a statistically significant effect on cluster prevalence, either in the form of wave energy, KeW (χ^2^_df = 6_ = 4.81, *p* = 0.569) or currents, KeC (χ^2^_df = 6_ = 9.00, *p* = 0.173). Finally, neither March (χ^2^_df = 6_ = 7.65, *p* = 0.265) nor August (χ^2^_df = 6_ = 9.40, *p* = 0.152) SST had a statistically significant effect on cluster prevalence.

### Structural equation modeling

**Figure 5:**
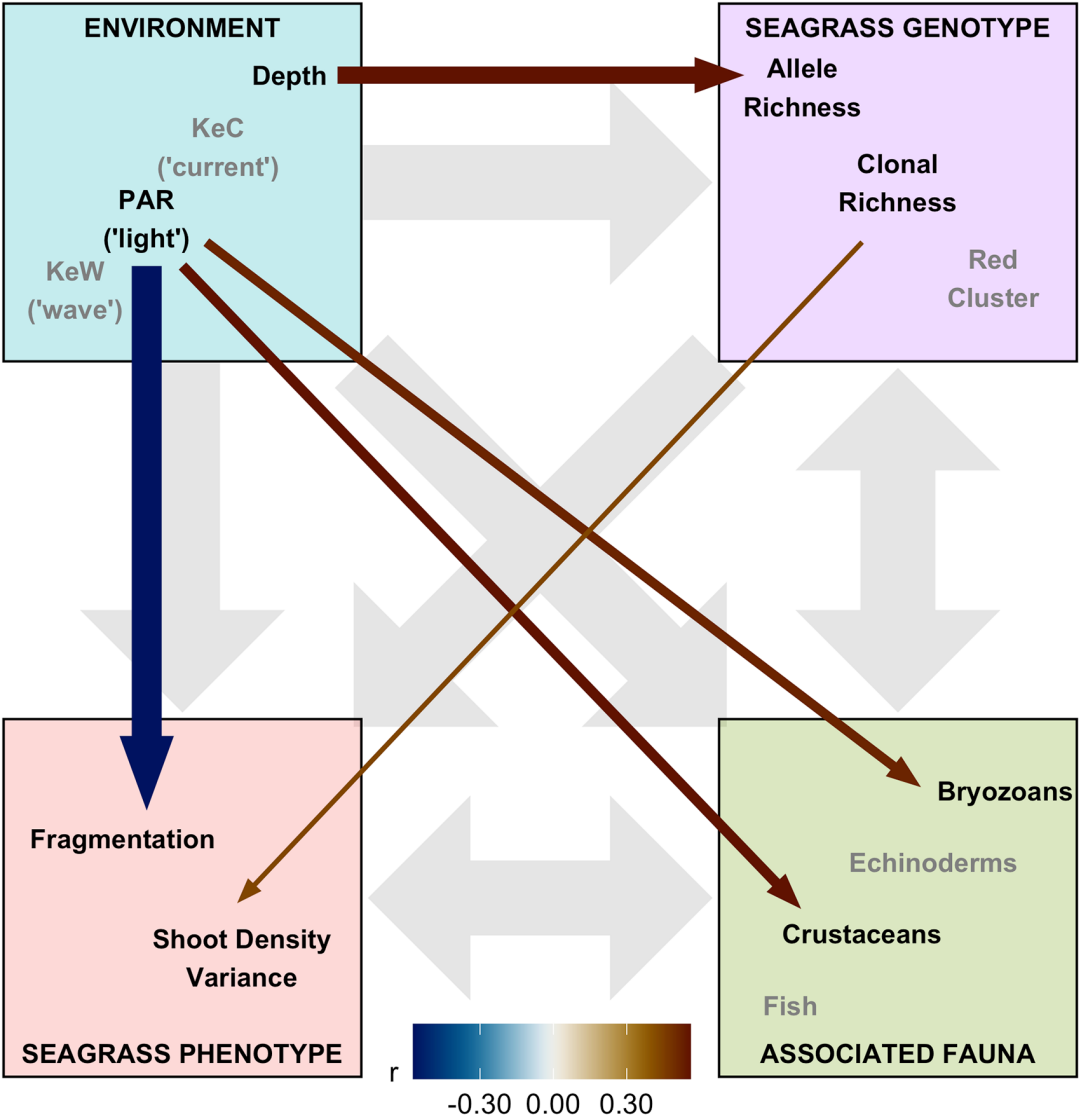
Structural equation model (SEM) schematic representation of *Zostera marina* ecosystem linkages from 17 locations across the south coast of England. Thirteen covariates were assigned to four ecosystem components: “environment,” “seagrass genotype,” “seagrass phenotype,” and “associated fauna.” Gray background arrows show assumed causal or bidirectional associations between ecosystem components. Covariates shown with black text were those found to have statistically significant associations with other components within our SEM (*p* < 0.01), and remaining covariates in the model shown with gray text. Colored foreground arrows show the magnitude and direction (−ve blue, red +ve) of associations, with arrow width scaled by statistical significance (smaller *p*-value shown as wider arrows).

Finally, we assembled “environment” (Depth, PAR, KeW, KeC, March and August SST), “seagrass genotype” [Ar, R, Fis, and genetic cluster (“red” and “blue,” with green having been removed through variance inflation factor analysis)], and “seagrass phenotype” metrics (quadrat level shoot density mean, variance, and presence/absence), along with data on “associated fauna” (nine groups, see Supplementary Figure 6), to test hypotheses on the network of relationships operating in this ecosystem. Pairwise Spearman rank correlation identified 16 of these 23 covariates had statistically significant (*p* < 0.05) correlations greater than *r* = 0.5 with variables in different ecosystem components (Supplementary Figure 6). SST and the “blue” genetic cluster were removed due to high correlation with KeW and Ar, respectively. The remaining 13 variables were included in our SEM and statistically significant (*p* < 0.01) linkages quantified in Figure 5. The SEM provided a very good fit to the data: χ^2^_df = 13_ = 13.8, *p* = 0.386 (a non-significant *p*-value indicates good SEM fit); GFI = 0.88 (analogous to R^2^); RMSEA = 0.06 (small indicates good fit); CFI = 0.97 (close to 1 indicates good fit).

Overall, we found the strongest link was a negative effect of light (PAR) on seagrass fragmentation [Estimate (SE) = −0.573 (0.167), z = −3.43, *p* < 0.001]. Here, higher fragmentation is defined as a smaller proportion of quadrats with seagrass present at a given location (as (Smale et al., 2019) from which fragmentation data are sourced): more light is associated with more continuous seagrass coverage. PAR also had positive links to abundance of bryozoans [Estimate (SE) = 0.520 (0.191), z = 2.72, *p* = 0.007] and crustaceans [Estimate (SE) = 0.560 (0.203), z = 2.76, *p* = 0.006]. As seen in Figure 3, we also recovered the positive association between depth and allelic richness, Ar, [Estimate (SE) = 0.561 (0.182), z = 3.08, *p* = 0.002] in our SEM (Figure 5). Therefore, “environmental” covariates had links with all three other components that we defined: “seagrass genotype,” “seagrass phenotype,” and “associated fauna.” The only other statistically significant link in our SEM was a positive association between seagrass clonal richness, R, and between-quadrat variance in seagrass shoot density [Estimate (SE) = 0.435 (0.169), z = 2.57, *p* = 0.010; Supplementary Figure 7].

## Discussion

Our study quantified variation and structure in seagrass genotype at 17 sampling locations across the south coast of the United Kingdom. We showed that this structure is associated with both environmental and geographic parameters. However, we present evidence that environment, more than genetics, predicts *Zostera marina* phenotypic variation and its associated faunal diversity. While our findings represent a substantial observational study of an important ecosystem in a natural setting, our analysis is correlative rather than mechanistic. In particular, the use of neutral loci (microsatellites) does not allow a test of whether phenotype in general is genetically versus environmentally determined. We go on to discuss our findings in that context.

We observed population genetic structuring at the regional scale in the form of isolation by distance (IBD). Isolation by distance has been observed in *Z. marina* previously across many areas of the northern hemisphere (Olsen et al., 2004; Muñiz-Salazar et al., 2005). At scales below c. 150 km, IBD was shown to be highly variable between location but displayed a clear “break point” at c. 150 km, above which IBD was consistently observable (Olsen et al., 2004). Our study was somewhat at odds, with a linear, or even decelerating pattern of IBD, up to a maximum of 170 km separation. However, the statistical evidence for nonlinearity in our study was weak, and the core finding of IBD is consistent with other studies and populations. Our study area consists of a series of suitable locations, separated by stretches of coastline where seagrass cannot survive, which would be expected to maximize potential for genetic differentiation with distance.

In addition, we observed population genetic structure at the local scale, with up to three genetic clusters at many sampling locations. In particular, through cluster analysis we found a genetic cluster strongly associated with estuarine environments. We are not aware of other reports of this type of population differentiation, but our findings are statistical and based on neutral markers. It would be interesting to follow up these findings with a trait-based approach, attempting to validate this pattern and infer the causal mechanism if upheld. Elsewhere, it has been found that *Z. marina* population genetics differ along a salinity gradient (Martínez-García et al., 2021) and that isolated locations such as fjords can harbor distinct genotypes (Olsen et al., 2013). It is also known from reciprocal transplant and common garden experiments that local *Z. marina* populations can show a “home-site advantage” at scales of a few kilometers (Hämmerli and Reusch, 2002; DuBois et al., 2022). Therefore, it may not be surprising to find seagrass genotypes selected for estuarine environments, and studies like ours, based on microsatellites, will hopefully provide support for whole genome approaches designed to investigate adaptation and selection.

To investigate species-habitat associations in our coastal ecosystem, we selected uncorrelated environmental variables likely to impact on *Z. marina* morphology, diversity, and resilience (Salo et al., 2015; Bertelli and Unsworth, 2018; Bertelli et al., 2021; Martínez-García et al., 2021). These broadly fell into three categories: light-associated, kinetic energy-associated, and sea surface temperature (SST). Kinetic energy may have direct impacts on seagrass through mechanical damage or act indirectly by increasing turbidity. In our data, we found low correlation between light and kinetic energy, so infer that any energy-mediated effects are mechanical here. We did not include some other environmental variables known to limit seagrass distribution, such as salinity (Martínez-García et al., 2021) and organic matter (Krause-Jensen et al., 2011), primarily because reliable data were not available at the locations and scales we needed. Both are known to affect *Z. marina* genetics (Martínez-García et al., 2021) and distribution (Krause-Jensen et al., 2011) and the potential for interactions between these and other environmental variables would be an interesting future research direction. We also included sea temperature in our analysis, as it is well known to affect seagrass growth (Blok et al., 2018; Hammer et al., 2018) and other regulatory processes of *Z. marina* such as host-pathogen interactions (Bull et al., 2012). However, sea surface temperature was strongly correlated with wave energy in our study, so we did not find evidence of direct correlations between temperature and other variables. Given the narrow latitudinal range of our survey locations, the main driver of differences in temperature is likely to be location within estuaries versus open coastline. This would account for the correlation with wave energy and might also be expected to be correlated with salinity. Finally, it should be noted that seagrass and associated fauna were sampled over August of two consecutive years and pooled. This survey design limitation was addressed by considering whether environmental variables were likely to vary substantially between 2 years. With the exception of SST, all the environmental data we used were obtained from databases that remain unchanged from year to year, based on the assumption that these variables are slow moving or static. In the case of SST, we demonstrated a strong positive correlation between years and calculated the variance inflation factor associated with this correlation to support retaining 2016 over 2017 SST. This is also the more appropriate year to retain on mechanistic grounds, being at the start of our survey period.

We found that our first environmental variable category (photosynthetically active radiation (PAR) and depth) had significant effects on all three ecosystem components: seagrass genotype, seagrass phenotype, and associated biodiversity. Our mixed effects modeling indicated increasing depth is associated with increasing allelic richness (Ar) but plateauing or possibly reversing at the deepest locations. In our study area, depths ranged between 1 and 4 m below chart datum, which represents the shallower end of the natural range in this region, with seagrass found down to around 10 m at some locations (Jackson et al., 2011). This may explain why we only identified weak evidence for the reversal of allele richness at the deeper end of our range. Elsewhere, *Z. marina* allelic richness has found to be maximized at intermediate depths along sub-tidal gradients (Hays et al., 2021), broadly consistent with our findings. Some other studies have appeared to show the opposite trend, with highest diversity at the shallowest depths, but these compared inter-tidal and sub-tidal meadows, so qualitatively different environments (Ruckelshaus, 1998; Kamel et al., 2012; Reynolds et al., 2017). While we do not probe mechanisms in our observational field study, the relationship between flowering versus clonal reproduction and depth could potentially explain our findings. However, the relationship between environment, flowering, and genetic diversity in *Z. marina* is complex and remains a key challenge to understand, as discussed by Hays et al. (2021).

Structural equation modeling confirmed our finding of increasing allelic richness with depth and showed that increasing light (PAR) was associated with less fragmented (more continuous) seagrass vegetation, as well as increased abundance of associated biodiversity (crustaceans and bryozoans). Overall, the positive effect of light on seagrass spatial continuity was the strongest ecosystem link identified in our study. Light is well known to be limiting to seagrass growth, but this is typically measured in terms of growth rate, shoot morphology, or shoot density (Grice et al., 1996; Collier et al., 2016; Bertelli and Unsworth, 2018) rather than heterogeneity in cover, or fragmentation. In fact, light was not associated with variation in shoot density in our study, suggesting reduced fragmentation in higher light conditions may not be simply through increased growth rate. However, light is known to promote flowering shoot production (van Lent et al., 1995), reproduction by seeds (as opposed to vegetative reproduction by rhizome extension) is associated with infilling of gaps in seagrass populations (Potouroglou et al., 2014), and vegetation fragmentation dynamics are best explained by infilling of gaps rather than localized losses (Irvine et al., 2016), providing a mechanism to explain our findings.

While kinetic energy due to waves (KeW) was weakly positively associated with increasing allele richness in our mixed effects modeling, this was not recapitulated in our SEM. We conclude that there is little evidence for kinetic energy-associated effects in our data, although all our study locations were relatively sheltered. As a further confounding issue, it is well known that seagrasses act as ecosystem engineers, attenuating wave energy and reducing turbidity (increasing ambient light), so self-facilitating its own growth (van der Heide et al., 2007; Adams et al., 2016; Reidenbach and Thomas, 2018). Since our sampling was typically conducted toward the center of seagrass meadows, mechanical stress from wave and current action may have been dampened below levels where variation drives observable effects on seagrass ecosystem properties.

Despite the population genetics structure evident in our study through analysis of molecular variance (AMOVA), local clustering, and IBD, we found little evidence to suggest that genetic and genotypic diversity had any association with the abundance of the seagrass or its associated fauna. The exception to this was a statistically significant positive association between clonal richness and variance in shoot densities within sampling locations. Genotypic diversity is well known to increase variability in traits such as productivity or biomass in animal and plant species [reviewed in Hughes et al. (2008)], including *Z. marina* (Hughes et al., 2009). However, in our study this association between seagrass genotype and phenotype had no connections to other ecosystem components.

Our finding that seagrass phenotype had no direct association with fauna is at odds with the positive association between *Z. marina* shoot density and faunal diversity found by McCloskey and Unsworth (2015) and various earlier studies, summarized in Attrill et al. (2000). As discussed by Attrill et al. (2000), this association could simply be explained as a sampling effect, as “more” seagrass offers greater overall opportunity for researchers to encounter additional species, particularly epifauna. Looking beyond shoot density (Jackson et al., 2006) specifically assessed the relationship between mobile fauna and seagrass habitat heterogeneity and patchiness (so niche diversity), with positive results. Again, this result is at odds with our results for shoot density variance and fragmentation. We see two possible explanations for our differing findings. A substantive difference between our study and those is that we assembled relatively coarse taxonomic data, while others have focused on higher taxonomic resolution but within restricted groups, such as fish or arthropods, and reduced spatial extent. Therefore, it could be that by accepting the trade-off between the amount versus resolution of data available through a large-scale citizen science project, we missed the appropriate taxonomic scale at which habitat-biodiversity relationships exist. Alternatively, our study differs from those in testing links between environment, seagrass genotype, seagrass phenotype, and associated biodiversity using an integrated SEM approach. Our model suggests that associations between seagrass complexity and faunal diversity may simply be correlative, driven by shared environmental drivers. These would otherwise be hard to disentangle either statistically or experimentally due to the positive feedback relationship between seagrass structure and turbidity, as well as sedimentation rates (Potouroglou et al., 2017). To partially resolve this, we note that the previously published analysis of the seagrass phenotype and associated fauna data in our study did report a positive relationship between fauna and both *Z. marina* density and continuity (Smale et al., 2019), suggesting that these faunal data are of sufficient resolution, and that analyses suited to quantifying linkages within high-dimensional ecosystems can play an important part in understanding complex field studies. Elsewhere, experimental approaches have been conducted, with a threshold effect observed such that faunal diversity is only strongly affected by substantial (e.g., > 50%) seagrass removal (Reed and Hovel, 2006) and that more specific aspects of seagrass fragmentation, such as distance to patch edges, explains epifauna diversity patterns (Moore and Hovel, 2010), suggesting that edge effects more than niche diversity *per se* might be the underlying driver of associated biodiversity.

Overall, we found relatively low levels of genetic diversity across our study, compared with (Oetjen et al., 2010), who developed the microsatellite panel we used. In our study, mean allele richness was 2.1, compared to 5.0 (calculated from the subset of their loci used in our study—see their Table 1) based on a comparable number of samples (284) from six locations at a maximal distance of 53 km apart across the Wadden Sea. This lack of genetic diversity may be the reason behind failing to find an association between genotype and other ecosystem components. However, genetic diversity is predicted to be lower at the leading edge of an expansion, e.g., post-glacial, compared to locations at the center of a species’ range or in ancient refugia, and this pattern has been documented in terrestrial [reviewed in Hewitt (2000)] and marine [reviewed in Maggs et al. (2008)] species across the north Atlantic, including in *Z. marina* (Diekmann and Serrao, 2012). Therefore, our finding that genetic diversity is poorly connected to other ecosystem components (seagrass phenotype and associated biodiversity) may be commonplace in other areas at the front of range expansion, or where genetic diversity is otherwise reduced, e.g., as part of a restoration program (Jahnke et al., 2015).

Alternatively, contemporary population genetic diversity and structure may be a recent historical legacy. This may not be surprising, given that much of current distribution of seagrass is a result of intense pressures and declines over the last hundred years (Short et al., 2006; Waycott et al., 2009), but that many of those pressures (e.g., wasting disease, industrial pollution) are considerably less severe now. Indeed, despite continuing seagrass losses globally, there is evidence that some populations in Europe may no longer be declining (de los Santos et al., 2019).

## Conclusion

To conclude, we found clear evidence of local environmental conditions being associated with variation in seagrass genotype, seagrass phenotype, and associated fauna, but little evidence of links between contemporary population genetic structure and ecosystem state: the environment, particularly ambient light, more than genetics predicts natural variation in other components of this temperate seagrass ecosystem. Many of our individual findings (genetic isolation by distance, an association between genotypic and phenotypic diversity, and the key role of light on seagrass distribution) are largely in agreement with other studies. However, by assembling these elements into a single analysis, we provide new insights into the relative roles of associations between ecosystem components. While it is undoubtedly true that even apparently simple ecosystems, such as seagrasses, comprise interesting and important complexities in their dynamics, understanding the dominant role played by predictable environmental drivers can help to focus conservation and restoration efforts.

## Data availability statement

The datasets presented in this study can be found in online repositories. The names of the repository/repositories and accession number(s) can be found in the article/ Supplementary material.

## Author contributions

JB and NA contributed to study design and data analysis. MP and JM coordinated sample collection. NA, EK, and RA-Q contributed to molecular data acquisition. CB and NA contributed to environmental data acquisition. All authors contributed to the article and approved the submitted version.

## Funding

This work was supported by the UK Natural Environment Research Council (NE/V016385/1) as part of the Sustainable Management of Marine Resources (SMMR) initiative awarded to JB, as well as a PhD scholarship awarded to NA by the Cultural Bureau of Saudi Arabia, with additional funding to NA from Princess Nourah bint Abdulrahman University, KSA. The Community Seagrass Initiative (CSI) project was supported by the UK Heritage Lottery Fund.

## Conflict of interest

The authors declare that the research was conducted in the absence of any commercial or financial relationships that could be construed as a potential conflict of interest.

## Publisher’s note

All claims expressed in this article are solely those of the authors and do not necessarily represent those of their affiliated organizations, or those of the publisher, the editors and the reviewers. Any product that may be evaluated in this article, or claim that may be made by its manufacturer, is not guaranteed or endorsed by the publisher.

## Footnotes

^1^ https://www.emodnet.eu/en/seabed-habitats

^2^ http://marine.copernicus.eu

^3^ https://emodnet.ec.europa.eu/en

^4^ http://marine.copernicus.eu
