# Erratum: Environment predicts seagrass genotype, phenotype, and associated biodiversity in a temperate ecosystem

**DOI:** 10.3389/fpls.2022.1035180

**Published:** 2022-10-03

**Authors:** Frontiers Production Office

**Affiliations:** Frontiers Media SA, Lausanne, Switzerland

**Keywords:** microsatellites, population genetics, *Zostera marina*, seagrass, coastal resilience

Due to a production error, **Figures 2**–**5** were published in the wrong order.

**Figure 2:**
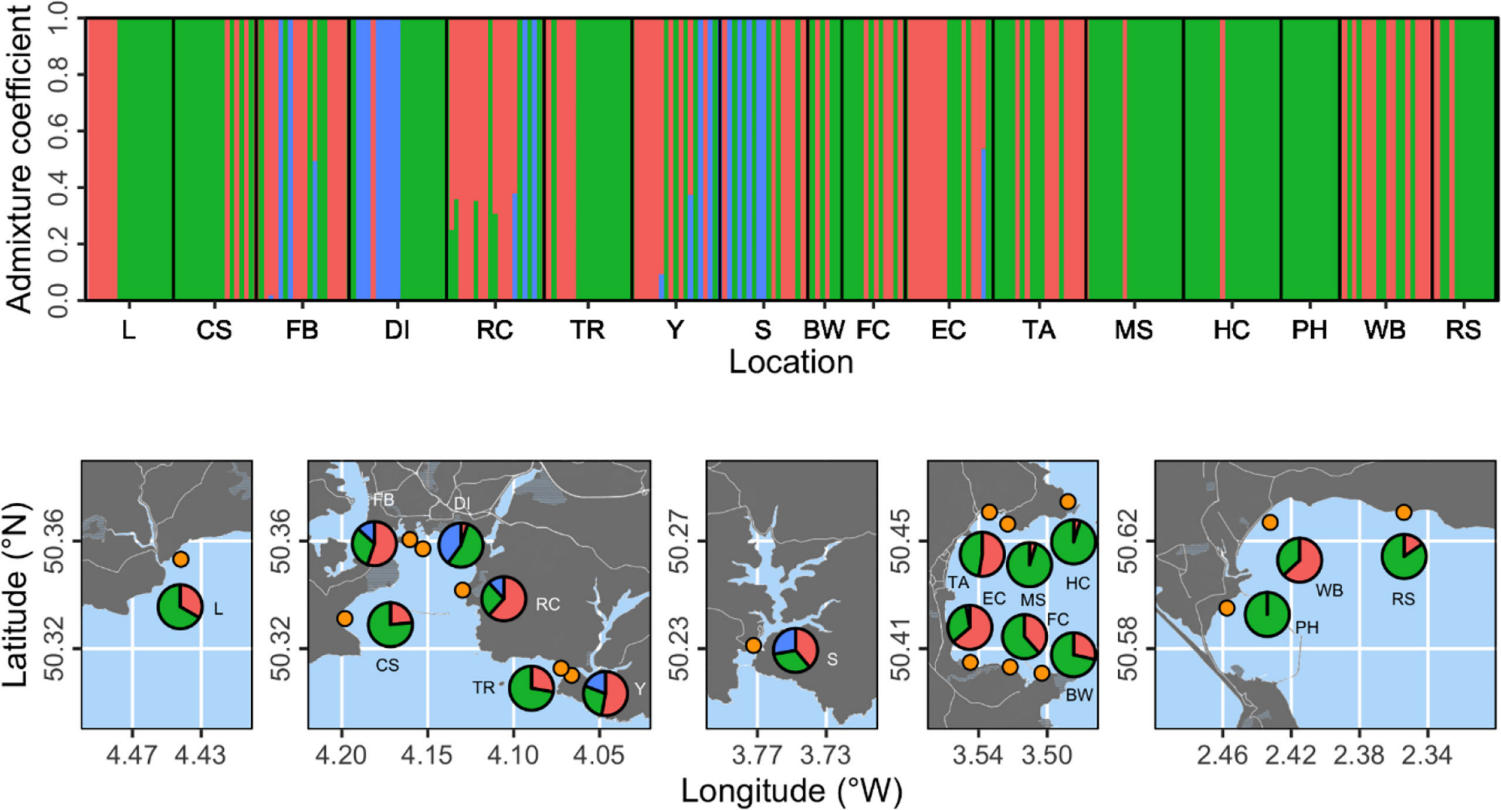
Population structure analysis based on three proposed genetic clusters. The top panel shows admixture coefficients indicating the probability of individual samples belonging to each of the three clusters (here shown as red, green, or blue). Bottom panels show location-level summaries of admixture coefficients at each of the 17 sampling locations (orange points).

**Figure 3:**
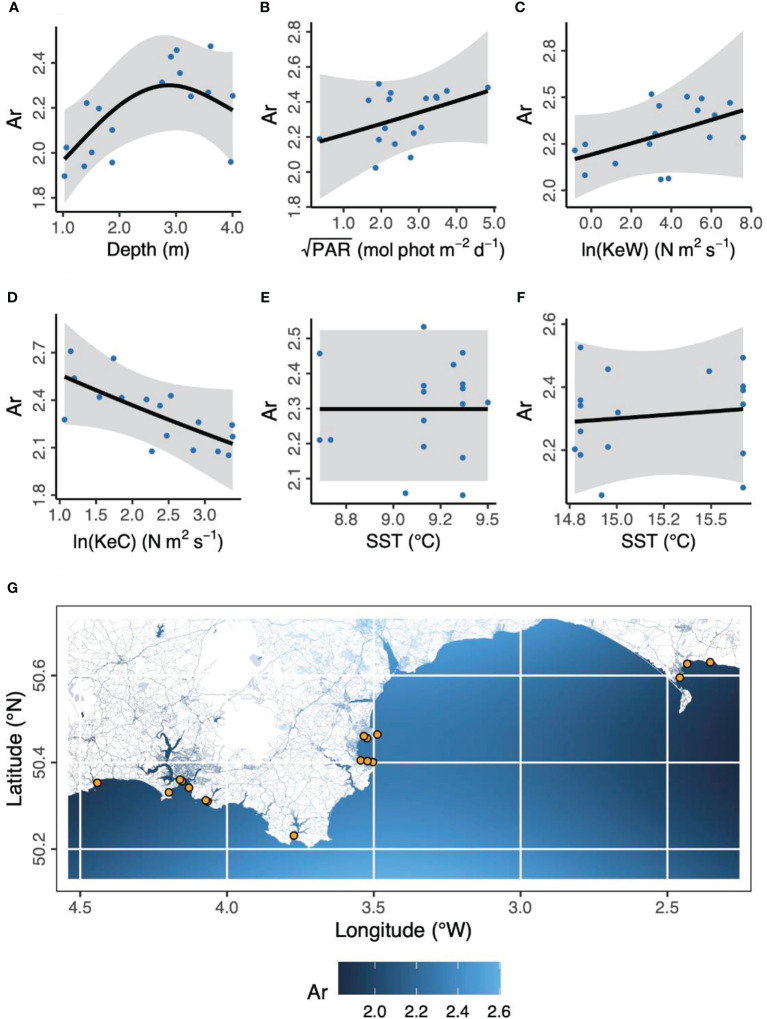
Environmental and geographical predictors of *Zostera marina* allelic richness, Ar, at 17 locations (orange points in panel **G**) across the south coast of England. **(A)** Depth is below chart datum. **(B)** PAR is photosynthetically active radiation at the seabed. KeW **(C)** and KeC **(D)** are kinetic energy, associated with waves and currents, respectively. SST is sea surface temperature in March **(E)** and August **(F)** 2016. Shaded ribbons show 95% confidence intervals and blue points are partial residuals. **(G)** The background color scheme represents fitted estimates of Ar in geographic space. Empirical values are shown in **Table 1**.

**Figure 4:**
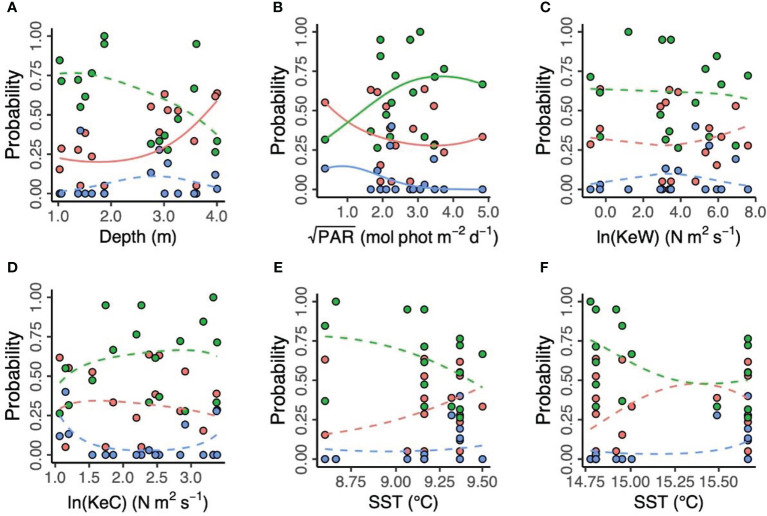
Environmental predictors of three putative *Zostera marina* clusters (“red,” “green,” and “blue”) in 17 locations across the south coast of England. **(A)** Depth is below chart datum. **(B)** PAR is photosynthetically active radiation at the seabed. KeW **(C)** and KeC **(D)** are kinetic energy, associated with waves and currents, respectively. SST is sea surface temperature in March **(E)** and August **(F)** 2016. Solid lines show statistically significant relationships (*p* < 0.05), with non-significant relationships shown as dashed lines.

**Figure 5:**
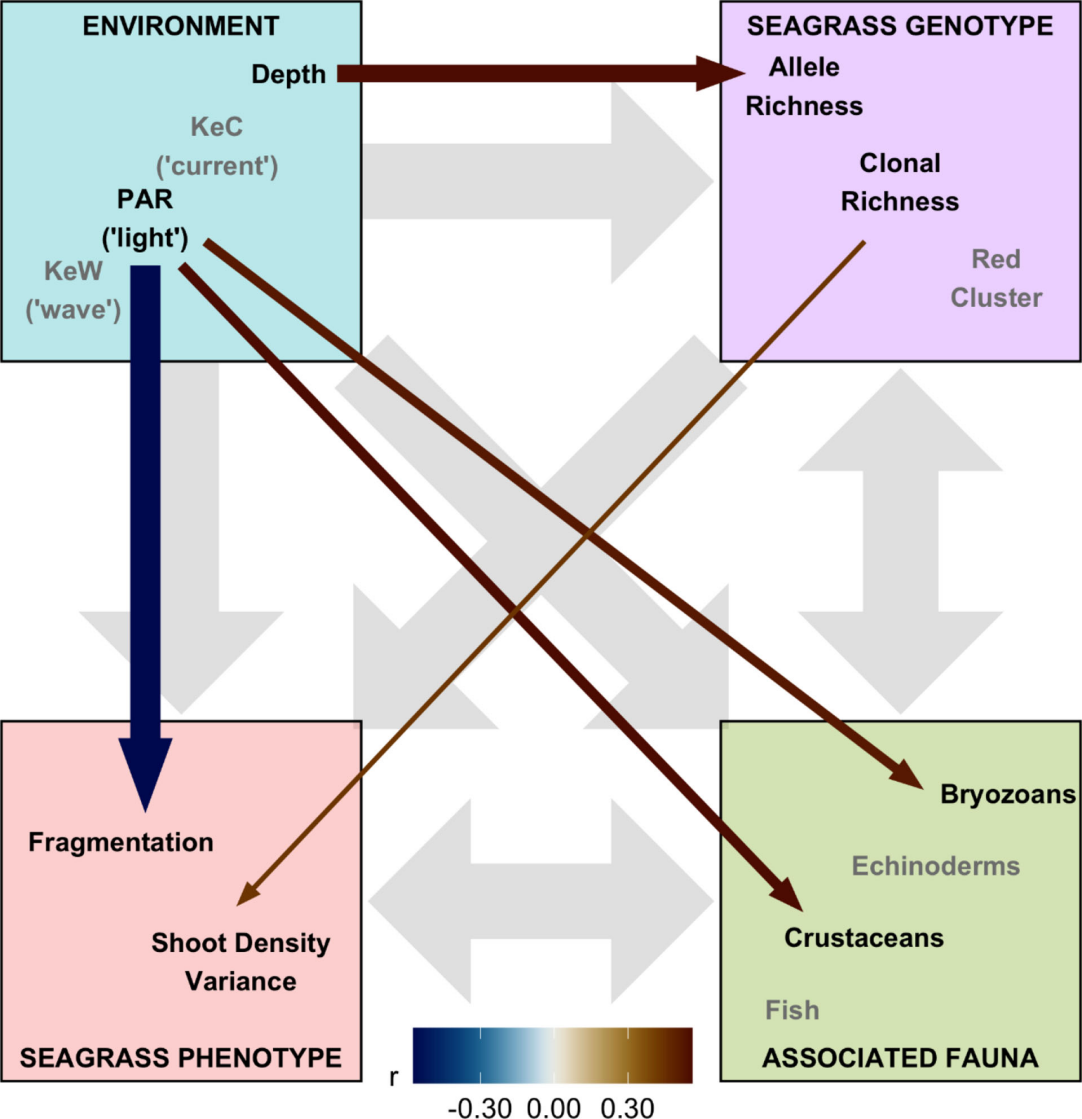
Structural equation model (SEM) schematic representation of *Zostera marina* ecosystem linkages from 17 locations across the south coast of England. Thirteen covariates were assigned to four ecosystem components: “environment,” “seagrass genotype,” “seagrass phenotype,” and “associated fauna.” Gray background arrows show assumed causal or bidirectional associations between ecosystem components. Covariates shown with black text were those found to have statistically significant associations with other components within our SEM (*p* < 0.01), and the remaining covariates in the model are shown with gray text. Colored foreground arrows show the magnitude and direction (−ve blue, red +ve) of associations, with arrow width scaled by statistical significance (smaller *p*-value shown as wider arrows).

The corrected figures and their captions appear below:

Moreover, since they were published in the wrong order, the figures were cited incorrectly in the **Results** section.

A correction has been made to the **Results** section, subsection **Environmental predictors of population genetics**, paragraph 1:

“In the case of allelic richness, Ar, (**Figure 3**) geographic space (longitude, latitude) was not statistically significant as fixed effects (Longitude, *F* = 0.902, *p* = 0.107; Latitude, *F* = 0.270, *p* = 0.185; Longitude × Latitude, *F* < 0.001, *p* = 0.534). We also found modeling space as autocorrelated random effects resulted in a worse model than no spatial structure (ΔAICc = 31.1). However, in ecological niche space (Depth, PAR, KeW, KeC, SST), we found an increase in Ar with increasing depth, plateauing by around 3 m below chart datum (*F* = 3.53, *p* = 0.030). We found no other statistically significant relationships between Ar and environmental covariates: light (PAR, *F* = 1.08, *p* = 0.326), kinetic energy due to waves (KeW, *F* = 1.25, *p* = 0.292), kinetic energy due to currents (KeC, *F* = 2.87, *p* = 0.124), and sea surface temperature (March SST, *F* < 0.001, *p* = 0.644; August SST, *F* = 0.073, *p* = 0.270).”

A correction has been made to the **Results** section, subsection **Environmental predictors of population genetics**, paragraph 4:

“Additionally, we modelled the effects of spatial and environmental predictors on the relative frequencies of each of the three proposed genetic clusters (**Figure 4**). We found no effect of longitude and latitude ( $\chi_{\text{df}=9}^{2}=12.39$ , *p* = 0.192, likelihood ratio test). Prevalence of the “red” cluster significantly increased with increasing depth (“Red,” $\chi_{\text{df}=2}^{2}=7.13$ , *p* = 0.028). However, depth did not have a statistically significant effect on prevalence of the “green” and (estuary-associated) “blue” clusters (“Green,” $\chi_{\text{df}=2}^{2}=4.46$ , *p* = 0.108; “Blue,” $\chi_{\text{df}=2}^{2}=1.35$ , *p* = 0.509). Light (PAR) had a statistically significant effect on all three clusters (“Red,” $\chi_{\text{df}=2}^{2}=42.1$ , *p* < 0.001; “Green,” $\chi_{\text{df}=2}^{2}=38.2$ , *p* < 0.001; “Blue,” $\chi_{\text{df}=2}^{2}=37.2$ , *p* < 0.001), with the “green” cluster more likely to be found in higher light conditions and the “red” and “blue” clusters more likely in lower light conditions. Kinetic energy did not have a statistically significant effect on cluster prevalence, either in the form of wave energy, KeW ( $\chi_{\text{df}=6}^{2}=4.81$ , *p* = 0.569) or currents, KeC ( $\chi_{\text{df}=6}^{2}=9.00$ , *p* = 0.173). Finally, neither March ( $\chi_{\text{df}=6}^{2}=7.65$ , *p* = 0.265) nor August ( $\chi_{\text{df}=6}^{2}=9.40$ ,*p* = 0.152) SST had a statistically significant effect on cluster prevalence.”

A correction has been made to the **Results** section, subsection **Structural equation modeling**, paragraph 1:

“Finally, we assembled “environment” (Depth, PAR, KeW, KeC, March and August SST), “seagrass genotype” [Ar, R, Fis, and genetic cluster (“red” and “blue,” with green having been removed through variance inflation factor analysis)], and “seagrass phenotype” metrics (quadrat level shoot density mean, variance, and presence/absence), along with data on “associated fauna” (nine groups, see Supplementary Figure 6), to test hypotheses on the network of relationships operating in this ecosystem. Pairwise Spearman rank correlation identified 16 of these 23 covariates had statistically significant (*p* < 0.05) correlations greater than *r* = 0.5 with variables in different ecosystem components (Supplementary Figure 6). SST and the “blue” genetic cluster were removed due to high correlation with KeW and Ar, respectively. The remaining 13 variables were included in our SEM and statistically significant (*p*< 0.01) linkages quantified in **Figure 5**. The SEM provided a very good fit to the data: $\chi_{\text{df}=13}^{2}=13.8$ ,*p* = 0.386 (a non-significant *p*-value indicates good SEM fit); GFI = 0.88 (analogous to R^2^); RMSEA = 0.06 (small indicates good fit); CFI = 0.97 (close to 1 indicates good fit).”

A correction has been made to the **Results** section, subsection **Structural equation modeling**, paragraph 2:

“Overall, we found the strongest link was a negative effect of light (PAR) on seagrass fragmentation [Estimate (SE) = −0.573 (0.167), z = −3.43, *p* < 0.001]. Here, higher fragmentation is defined as a smaller proportion of quadrats with seagrass present at a given location (as (Smale et al., 2019) from which fragmentation data are sourced): more light is associated with more continuous seagrass coverage. PAR also had positive links to abundance of bryozoans [Estimate (SE) = 0.520 (0.191), z = 2.72, *p* = 0.007] and crustaceans [Estimate (SE) = 0.560 (0.203), z = 2.76, *p* = 0.006]. As seen in **Figure 3**, we also recovered the positive association between depth and allelic richness, Ar, [Estimate (SE) = 0.561 (0.182), z = 3.08, *p* = 0.002] in our SEM (**Figure 5**). Therefore, “environmental” covariates had links with all three other components that we defined: “seagrass genotype,” “seagrass phenotype,” and “associated fauna.” The only other statistically significant link in our SEM was a positive association between seagrass clonal richness, R, and between-quadrat variance in seagrass shoot density [Estimate (SE) = 0.435 (0.169), z = 2.57, *p* = 0.010; Supplementary Figure 7].”

The publisher apologizes for these mistakes. The original version of this article has been updated.

In addition to these production errors, the authors identified errors in one of the affiliations and the funding statement.

In the original article, affiliation 2 was written incorrectly. Instead of “Department of Biology, Princess Nourah Bint Abdulrahman University, Riyadh, Saudi Arabia”, it should have been written as “Department of Biology, Princess Nourah bint Abdulrahman University, Riyadh, Saudi Arabia”.

Furthermore, a funder was mistakenly omitted from the funding statement. The funding statement has been corrected to:

“This work was supported by the UK Natural Environment Research Council (NE/V016385/1) as part of the Sustainable Management of Marine Resources (SMMR) initiative awarded to JB, as well as a PhD scholarship awarded to NA by the Cultural Bureau of Saudi Arabia, with additional funding to NA from Princess Nourah bint Abdulrahman University, KSA. The Community Seagrass Initiative (CSI) project was supported by the UK Heritage Lottery Fund.”

The authors apologize for these errors and state that these do not change the scientific conclusions of the article in any way. The original article has been updated.

