# Supplementary material for: Environment predicts seagrass genotype, phenotype, and associated biodiversity in a temperate ecosystem
Source: Front Plant Sci. 2022 Aug 4;13:887474. doi: 10.3389/fpls.2022.887474 (PMC9386313; doi:10.3389/fpls.2022.887474)
Supplement: Supplementary file 1 [file Data_Sheet_1.pdf]

Environment predicts seagrass genotype, phenotype, and associated biodiversity in a temperate ecosystem

Nahaa M Alotaibi, Emma J Kenyon, Chiara M Bertelli, Rahmah N Al-Qthanin, Jessica Mead, Mark Parry, James C Bull

Supplementary Material

Supplementary Table 1. Microsatellite primer sequences and fluorescent labelling (6-*FAM* or *HEX*) from Oetjen et al., 2010.

Locus name	Accession number	Primer sequences
CL766Contig1	FN435336	F:*FAM*-GAACGTTTCCCGGTCATTT<br>R:GGAATCGGTCAAGCAAAAAC
CL11Contig1	FN435337	F:*FAM*-GTGGAGGAAAGTGTGGGTGT<br>R:CTTGCATCCACCTTCATTTG
CL559Contig1	FN435338	F:*FAM*-CCACTTCCGTAGTTGCTGTT<br>R:CGATGAGGACGATGAGGAAT
ZME02125	FN435339	F:*FAM*-CGTTCAACTCAACACGCATT<br>R:GGTGACGAAAAGAAGCGAAG
ZMF02381	FN435340	F:*HEX*-GTGCAGGCGATCGAGTTATC<br>R:AAATTCGAGCTCTCAACTTCAA
CL202Contig1	FN435341	F:*FAM*-TTGAAAAGATTAATTATTGGTGGTG<br>R:TCAAGTCCGGATAAATTCGAT
CL380Contig1	FN435342	F:*FAM*-CCGCCTTCTTCTTCGTTAGA<br>R:TGTTGTTCTTGGAAAAGAATCAGT
CL805Contig1	FN435343	F:*HEX*-GGGGAGGTTTCCGAATACTTT<br>R:TGGAAGATGTTGGACATGGA
CL172Contig1	FN435344	F:*FAM*-CTCCTGGACGCAGAAATATG<br>R:GACAAACGATTAATTCAGAAACAAAA
CL53Contig1	FN435345	F:*FAM*-AACTCCTGGCGCAACTACTG<br>R:CTTCGTTTGCGGTTGCTT

ZME06302	FN435346	F:*FAM*-TCTAGCTTGTCGATGGCTGA<br>R:CCGTCAAATGTTTCCAAGGT
ZMC05062	FN435347	F:*HEX*-GAAGCCAACTTAATTCAACATCG<br>R:TTAATATAAATCCGAGACACAGACTC
ZMC19062	FN435348	F:*FAM*-CACTCTCCTCTTTCCGTTCG<br>R:CAGGGGCCTTCCTCTTACTC
ZME05315	FN435349	F:*FAM*-AAACGAGATGGTGGTTCCAT<br>R:TGCGAGCAGCTAACTAAGTCC
ZME02369	FN435350	F:*FAM*-AAGTCGAAATGGGGATACCA<br>R:TCGTCGGAAGAAAAAGAAGC

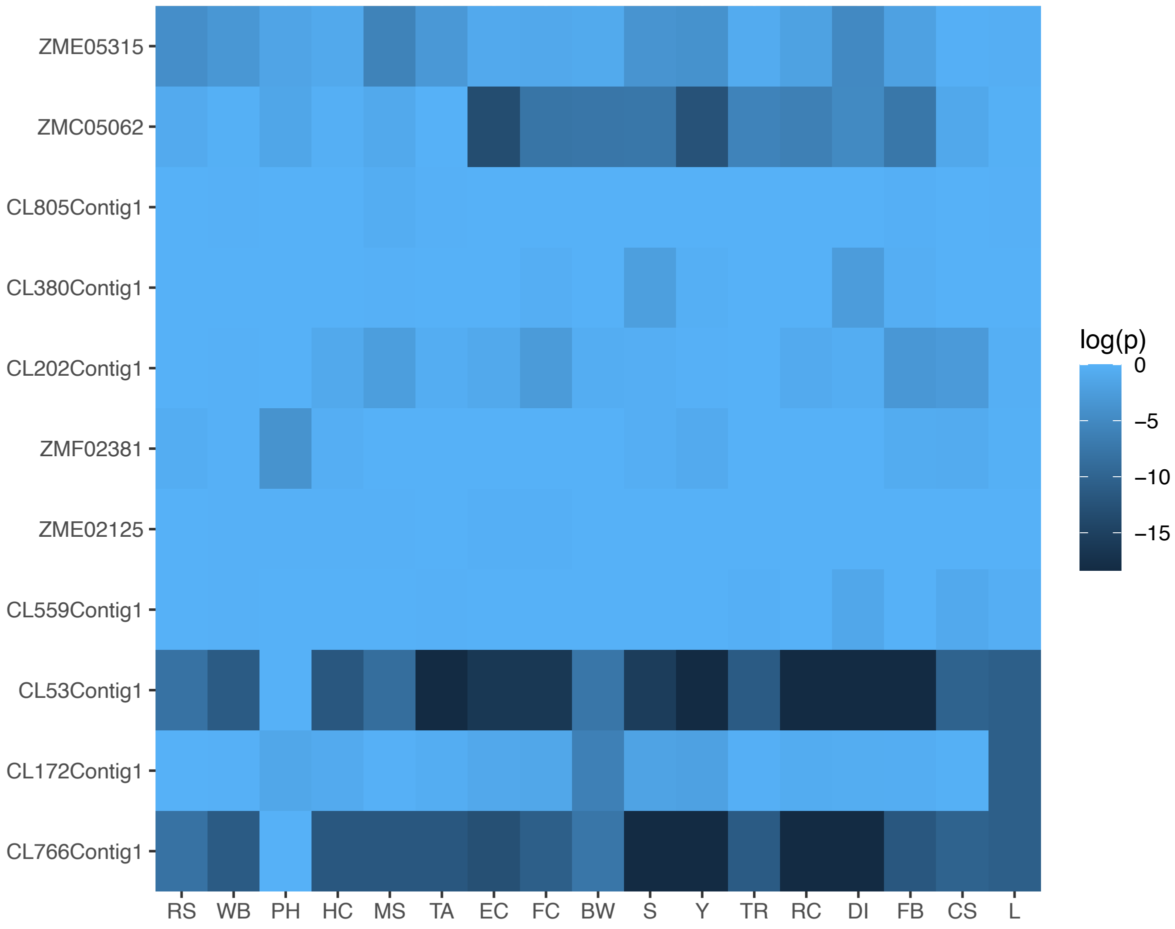

Supplementary Figure 1. Evidence for departure from Hardy-Weinberg equilibrium at 11 polymorphic microsatellite loci (rows) across 17 sampling locations (columns). Colour scale represents the natural logarithm of p-values.

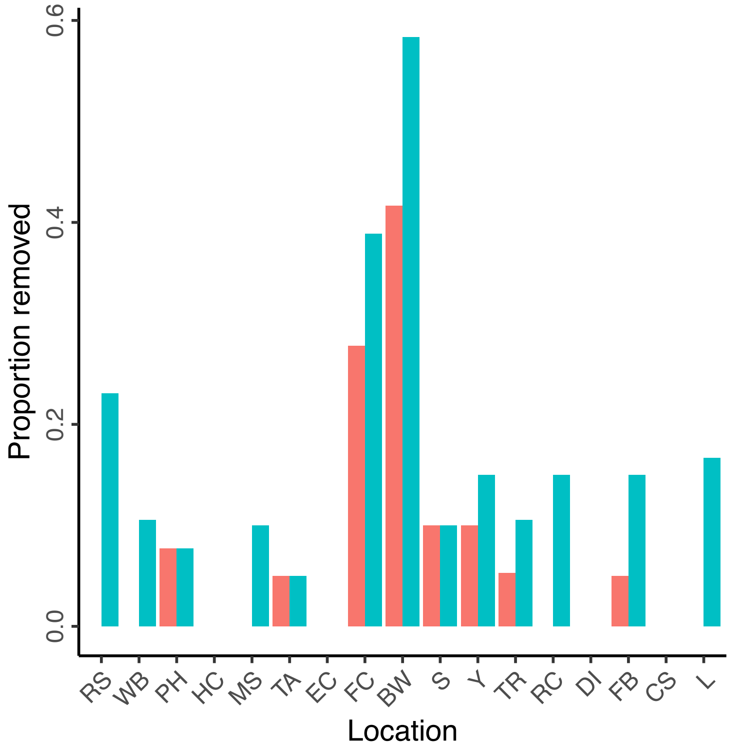

Supplementary Figure 2. The proportion of samples removed as having unacceptably low probability of having arisen through independent reproductive events, $P_{SEX} < 0.05$. This was performed based on the assumption that non-independent samples could (turquoise) or could not (orange) successfully relocate between the 17 sampling locations.

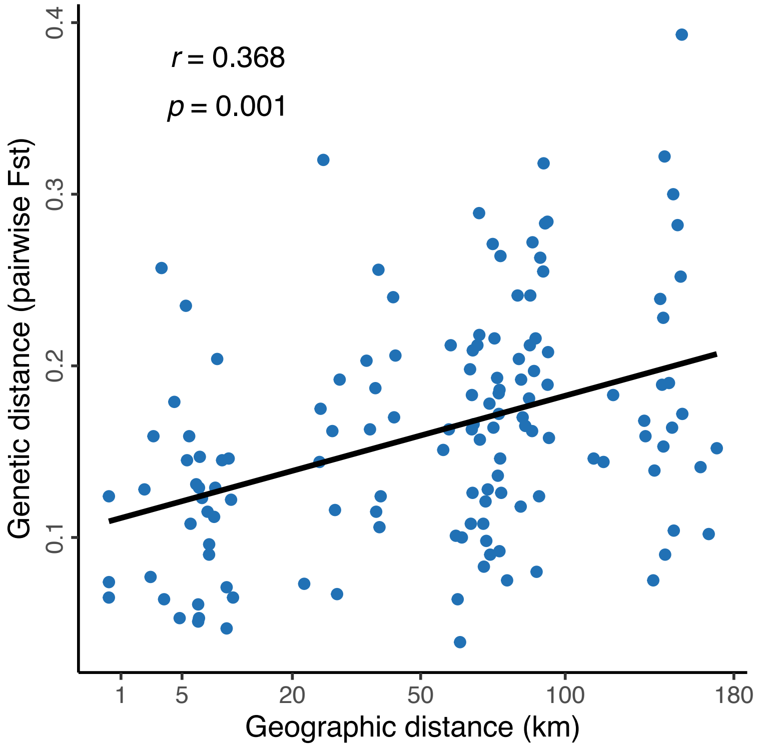

Supplementary Figure 3. Isolation by distance of pairwise Fst values across 17 sampling locations. Note the linear relationship on a square root scale. Inset statistical output shows the correlation, *r*, and the p-value, *p*, from a Mantel test.

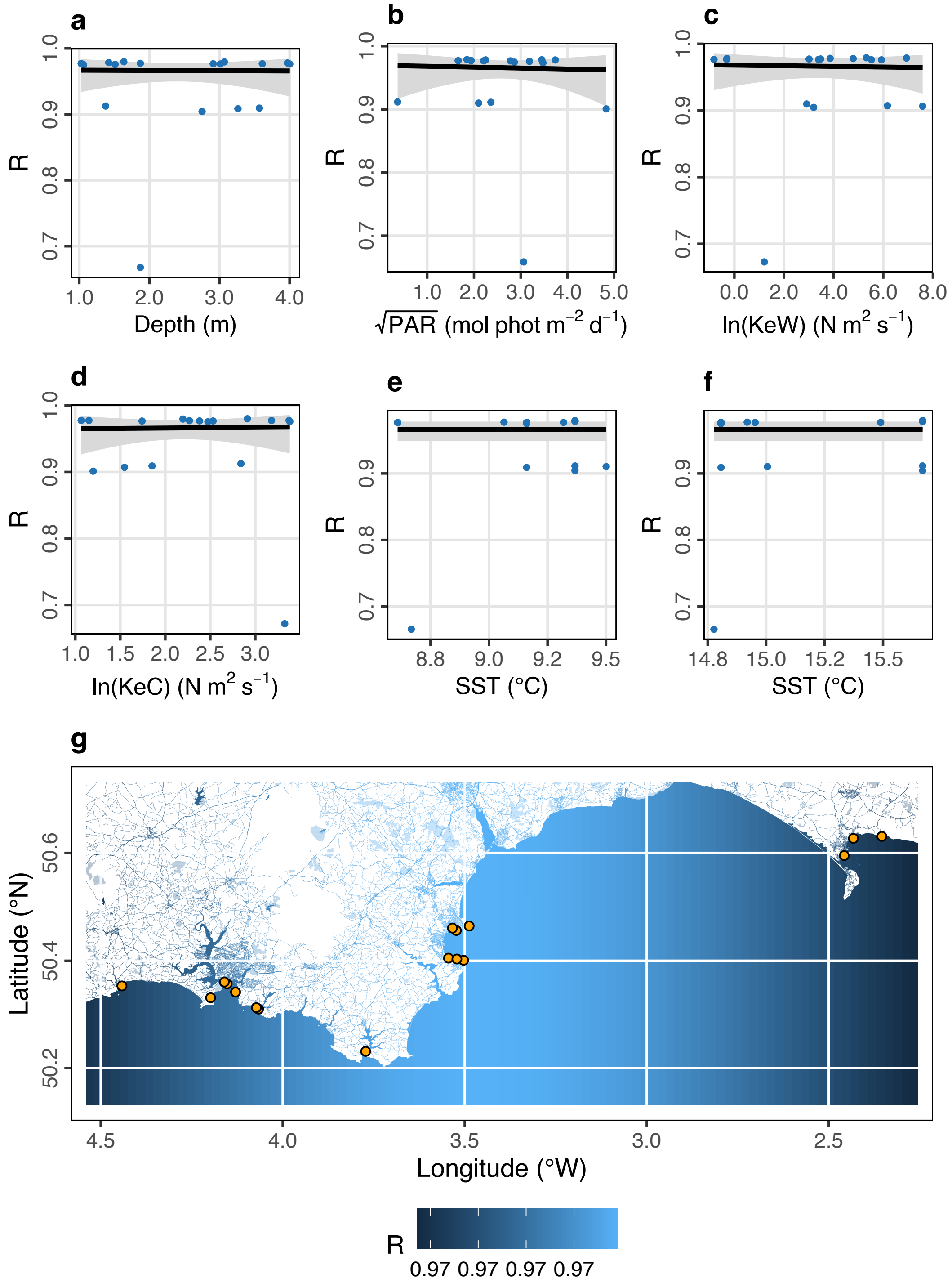

Supplementary Figure 4. Environmental and geographical predictors of *Zostera marina* clonal richness, R, at 17 locations (orange points in panel e) across the south coast of England. Panel a: Depth is below chart datum. Panel b: PAR is photosynthetically active radiation at the seabed. KeW (panel c) and KeC (panel d) are kinetic energy, associated with waves and currents respectively. SST is sea surface temperature in March (panel e) and August (panel f) 2016. Shaded ribbons show 95 % confidence intervals and blue points are partial residuals. In panel g, the background colour scheme represents fitted estimates of R in geographic space. Empirical values are shown in Table 1.

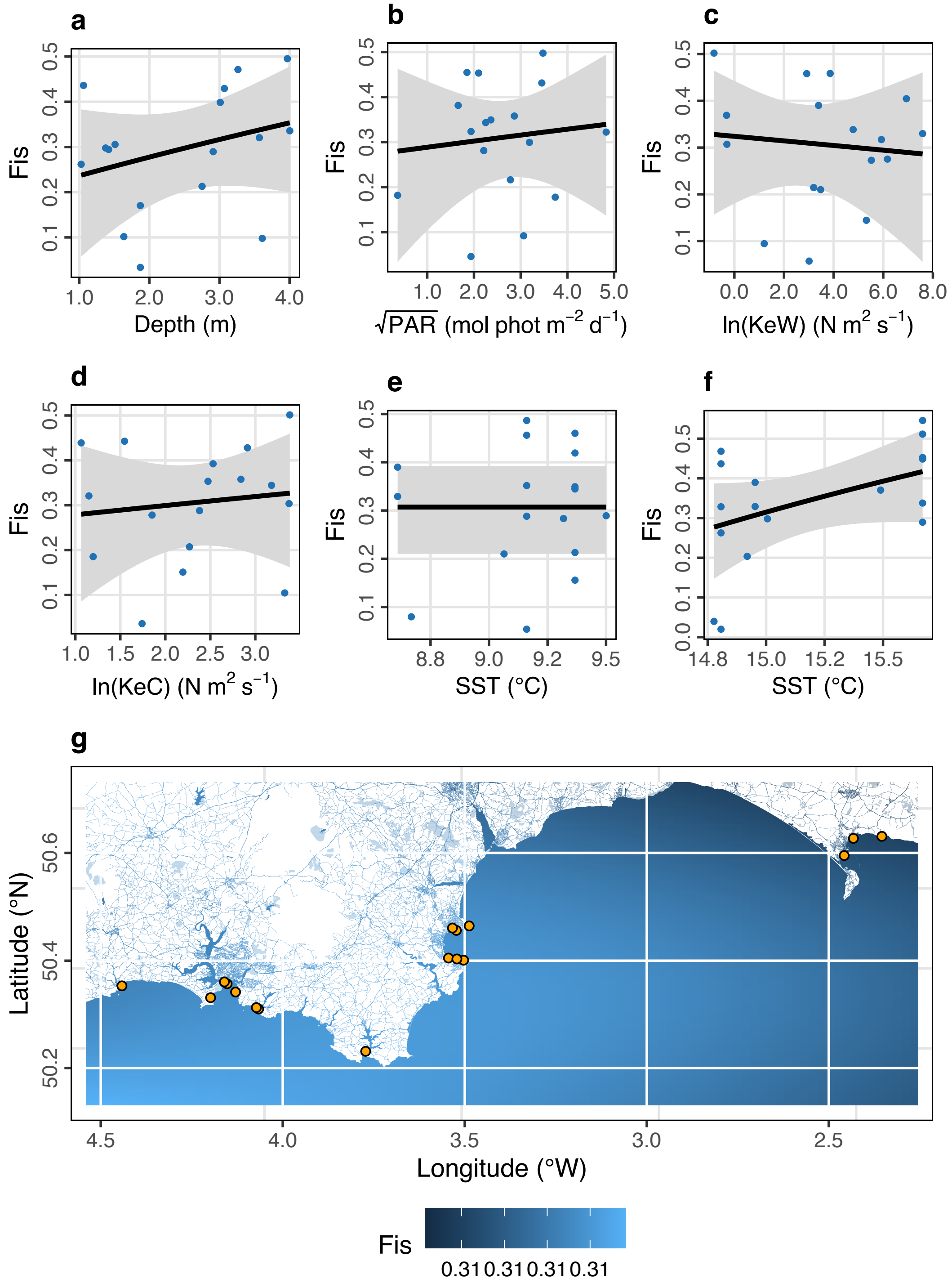

Supplementary Figure 5. Environmental and geographical predictors of *Zostera marina* coefficient of inbreeding, Fis, at 17 locations (orange points in panel e) across the south coast of England. Panel a: Depth is below chart datum. Panel b: PAR is photosynthetically active radiation at the seabed. KeW (panel c) and KeC (panel d) are kinetic energy, associated with waves and currents respectively. SST is sea surface temperature in March (panel e) and August (panel f) 2016. Shaded ribbons show 95 % confidence intervals and blue points are partial residuals. In panel g, the background colour scheme represents fitted estimates of R in geographic space. Empirical values are shown in Table 1.

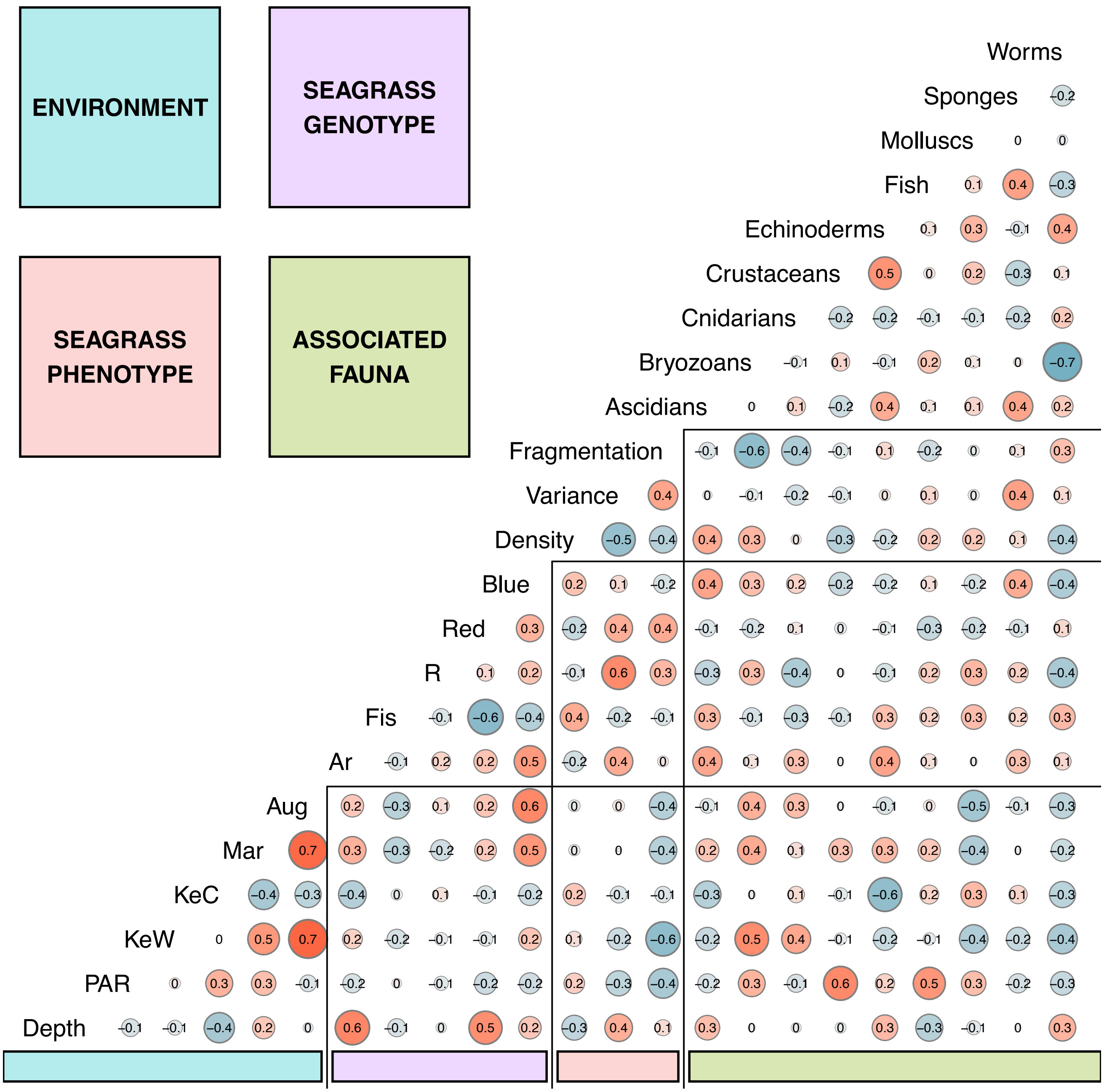

Supplementary Figure 6. Pairwise Spearman rank correlation values between environmental (turquoise), seagrass genotypic (magenta), seagrass phenotypic (orange), and associated fauna (green) variables. Circles are size-scaled by correlation value and colour-coded using a blue (-ve) to red (+ve) diverging colour scale. Red and Blue refer to the genetic clusters assigned those colours. Mar and Aug refer to mean sea surface temperature in March and August 2016.

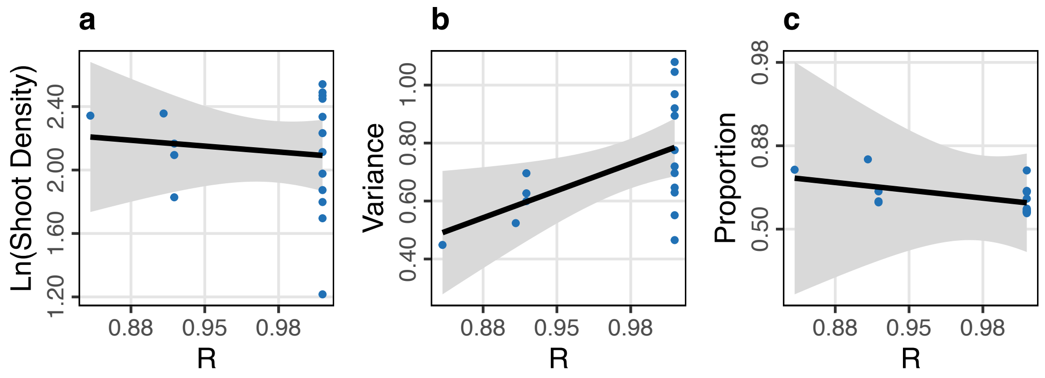

Supplementary Figure 7. Clonal richness, R, as a predictor of *Zostera marina* shoot phenotype: Mean ln(shoot density) per 0.25 m^2 quadrat (panel a), variance in ln(shoot density) per 0.25 m^2 quadrat (panel b), and the proportion of occupied 0.25 m^2 quadrats (panel c). Shaded ribbons show 95 % confidence intervals and blue points are partial residuals.
